# Scaling up and down of 3-D floating-point data in quantum computation

**DOI:** 10.1038/s41598-022-06756-w

**Published:** 2022-02-17

**Authors:** Meiyu Xu, Dayong Lu, Xiaoyun Sun

**Affiliations:** 1 School of Mathematics and Statistics, Henan University of Science and Technology, Luoyang, 471000 China; 2 School of Mathematics and Statistics, Henan University, Kaifeng, 475001 China; 3 College of Information and Management Science, Henan Agricultural University, Zhengzhou, 450000 China

**Keywords:** Quantum information, Computer science

## Abstract

In the past few decades, quantum computation has become increasingly attractive due to its remarkable performance. Quantum image scaling is considered a common geometric transformation in quantum image processing, however, the quantum floating-point data version of which does not exist. Is there a corresponding scaling for 2-D and 3-D floating-point data? The answer is yes. In this paper, we present a quantum scaling up and down scheme for floating-point data by using trilinear interpolation method in 3-D space. This scheme offers better performance (in terms of the precision of floating-point numbers) for realizing the quantum floating-point algorithms than previously classical approaches. The Converter module we proposed can solve the conversion of fixed-point numbers to floating-point numbers of arbitrary size data with $p+q$ qubits based on IEEE-754 format, instead of 32-bit single-precision, 64-bit double-precision and 128-bit extended-precision. Usually, we use nearest-neighbor interpolation and bilinear interpolation to achieve quantum image scaling algorithms, which are not applicable in high-dimensional space. This paper proposes trilinear interpolation of floating-point data in 3-D space to achieve quantum algorithms of scaling up and down for 3-D floating-point data. Finally, the quantum scaling circuits of 3-D floating-point data are designed.

## Introduction

Quantum computation is a theoretical computation system that performs operations on data by using quantum-mechanical phenomena (such as superposition and entanglement). In 1982, Feynman^1^ proposed the concept of quantum computer at first. After that, the integer factoring problem^2^ and database search algorithm^3^ were essential evidences supporting the power of quantum computers. Researches in quantum image processing field started with proposals on quantum image representations such as Qubit Lattice^4^, Real Ket^5^ and Entangled Image^6^. After 2011, some more practical quantum image models using entangled states (FRQI^7^) and superposition states (NEQR^8^, INEQR^9^, GQIR^10^, QRDS^11^, GFPRQS^12^, QR2-DD^13^, etc.) have been proposed. With the proposal of NEQR representation, Chetia et al.^14^ introduced a quantum image edge detection algorithm based on NEQR. Chakraborty et al.^15^ provided a brief survey of the current status of research in the quantum image processing paradigm in 2018. In addition, Chakraborty et al.^16^ proposed a combination of three different approaches for representing color quantum images in ternary quantum system. In 2018, Chakraborty et al.^17^ proposed and designed a circuit level implementation of the quantum multilevel threshold based color image segmentation technique.

At present, quantum image processing research has been paid more attention to the transform domain. Fijany and Williams^18^ studied fast algorithms and complete circuits for quantum wavelet transforms (QWT). Caraiman and Manta^19^ introduced quantum image filtering in the frequency domain. Quantum arithmetic operations were given by Ruiz-Perez and Garcia-Escartin^20^ based on quantum Fourier transform (QFT). Li and Sun^21^ presented quantum color image filtering in the frequency domain. There are many research results in the transform domain. Asaka et al.^22^ discussed an implementation of the fast Fourier transform (FFT) as a quantum circuit. The quantum version of the FFT (QFFT) is defined as a transformation of a tensor product of quantum states. Chakraborty et al.^23,24^ proposed the image denoising schemes based on QWT. Chang and Vasilakos^25^ presented core concepts of QFT and inverse QFT and given the reason of why QFT and inverse QFT are able to give exponential speed-up for FFT. Grigoryan and Agaian^26^ proposed a new quantum representation of images: Fourier transform representation. Indeed, the QFT is a key ingredient of many important quantum algorithms, including Shor’s factoring algorithm and the quantum phase estimation algorithm to estimate the eigenvalues of a unitary operator. Therefore, we propose the design scheme of quantum algorithm for 3-D floating-point data based on QFT in this paper.

In recent years, quantum image scaling as a kind of image geometric transformation has been widely studied and applied in quantum image processing. Jiang and Wang^9^ proposed quantum algorithms and circuits to realize the quantum image scaling based on the INEQR model for quantum images using the nearest neighbor interpolation. Jiang et al.^10^ given an improved quantum image representation GQIR and proposed a quantum algorithm to scale up quantum images based on nearest-neighbor interpolation with integer scaling ratio. Sang et al.^27^ constructed the quantum circuits of the nearest-neighbor interpolation method for FRQI and NEQR. Zhou et al.^28^ proposed the bilinear interpolation method for NEQR and given the corresponding quantum realization circuits. Li and Liu^29^ designed quantum image scaling using bilinear interpolation method based on QFT. Zhou et al.^30^ given quantum image scaling based on bilinear interpolation with arbitrary scaling ratio. However, as far as we know, three-dimensional (3-D) quantum floating-point data versions of which do not exist.

The problem of classical 3-D image (or data) reconstruction is currently a hot topic, such as the reconstruction technology of 2-D to 3-D data. We given a method to convert a 3-D data into 2-D data based on QR2-DD^13^. The dimensionality reduction from 3-D to 2-D data can reduce the number of qubits, and consequently, resources are saved at location information. As far as we know, the inverse problem, that is, 2-D to 3-D reconstruction problem, such as computed tomography (CT) imaging, has important practical significance. However, the quantum floating point data are not discussed. Inspired by this, we conducted preliminary explorations on the reconstruction of 2-D to 3-D quantum floating-point data. Therefore, the further research of our work can provide research ideas for the reconstruction of 2-D to 3-D quantum data.

Floating-point arithmetic precision is limited in length, such as the IEEE single (double, extended) precision format is 32-bit (64-bit, 128-bit) long. However, some problems require a longer floating-point format because of round-off errors. Such problems are usually solved in arbitrary precision (p+q)^13^.

Compared with fixed-point numbers, floating-point numbers offer great savings in the number of qubits when the required range of values or relative precision is large. It is very meaningful to consider the quantum scaling of floating-point data in 3-D space. Li and Liu^29^ designed quantum image scaling using bilinear interpolation method based on QFT, in which two core operations (i.e., addition and multiplication) are implemented using QFT. Therefore, we consider using trilinear interpolation method to study the quantum scaling of floating-point data in 3-D space. In this paper, we present a quantum scaling up and down scheme of floating-point data by using trilinear interpolation method based on QFT in 3-D space. It has three main contributions:

- The trilinear interpolation method for quantum scaling up and down of 3-D floating-point data is proposed for the first time.
- The generalized floating-point quantum representation of 3-D data in this paper can represent the arbitrary precision (p+q).
- A Converter module for converting fixed-point numbers to floating-point numbers is proposed.

In addition, based on QFT, we design the addition and multiplication (Q-Adder and Q-Multiplier modules) of 3-D floating-point data. Combining some basic modules in this paper, we propose the design scheme of the quantum scaling up and down of 3-D floating-point data using trilinear interpolation method based on QFT. Finally, we give the quantum scaling circuits of 3-D floating-point data.

The remainder of this paper is organized as follows: “Preliminaries’ ’section gives a brief introduction to quantum representation of 3-D floating-point numbers, classical interpolation methods and QFT. After the description of the basic modules in “Some modules” section, the addition and multiplication of the floating-point based on QFT (Q-Adder and Q-Multiplier) and Converter for converting fixed-point numbers to floating-point numbers are designed in “Floating-point addition and multiplication based on QFT” section. “Quantum scaling-up circuit for 3-D floating-point data” and “Quantum scaling-down circuit for 3-D floating-point data”sections introduce the design scheme of quantum scaling up and down for 3-D floating-point data using trilinear interpolation method. “Complexity analysis” section analyzes the computational complexity of the scaling circuits based on the elementary gates. Finally, conclusions and future research works are described in “Conclusions” section.

## Preliminaries

In this section, we briefly explain an overview of the foundation of the proposed methodology, including quantum representation of 3-D floating-point numbers, classical interpolation methods and QFT.

A quantum gate is simply an operator that acts on qubits. Such operators can be represented by unitary matrices. Some of the basic gates and their corresponding matrices are shown in (1). The identity gate (*I*), Hadamard gate (*H*), NOT gate (*X*), controlled-NOT gate (*CNOT*) and controlled-phase gate ($CR_k$) are well-known^31^, here,

1 $$\begin{aligned} I=\left( \begin{matrix} 1&{}0\\ 0&{}1 \end{matrix} \right) , H=\frac{1}{\sqrt{2}}\left( \begin{matrix} 1&{}1\\ 1&{}-1 \end{matrix} \right) , X=\left( \begin{matrix} 0&{}1\\ 1&{}0 \end{matrix} \right) , CNOT=\left( \begin{matrix} 1&{}0&{}0&{}0\\ 0&{}1&{}0&{}0\\ 0&{}0&{}0&{}1\\ 0&{}0&{}1&{}0 \end{matrix} \right) , CR_k=\left( \begin{matrix} 1&{}0&{}0&{}0\\ 0&{}1&{}0&{}0\\ 0&{}0&{}1&{}0\\ 0&{}0&{}0&{}e^{2\pi i/2^k} \end{matrix} \right) . \end{aligned}$$

Format of floating-point numbers in IEEE-754 (Institute of Electrical and Electronics Engineers, IEEE)^13^ is shown in Table 1.

**Table 1:** Format of floating-point numbers in IEEE-754.

*s*	*e*	*f*
*signbit*	*exponent*	*fractional*(*mantissa*)

### Generalized floating-point quantum representation of 3-D data

**Figure 1:**
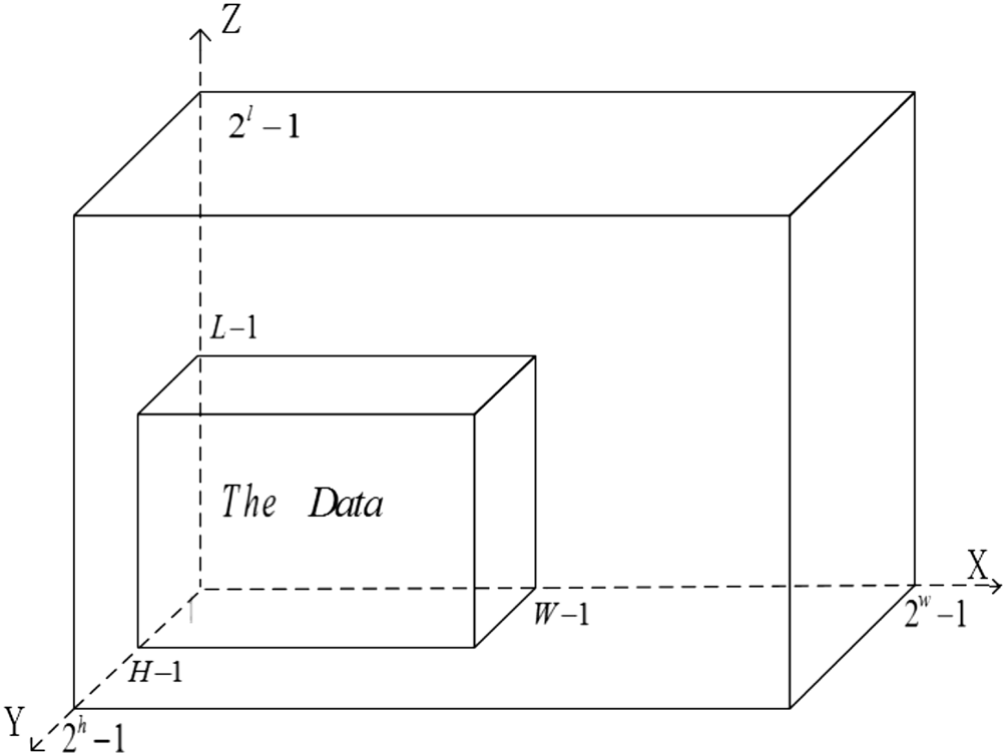
3-D data.

Suppose binary sequence $|s_{YXZ}^{0}s_{YXZ}^{1}\ldots s_{YXZ}^{p-1}s_{YXZ}^{p} s_{YXZ}^{p+1}\ldots$ $s_{YXZ}^{p+q-1}\rangle$ encodes the floating-point number $|S_{YXZ}\rangle _F$ of 3-D data corresponding to the location of $|YXZ\rangle$. This proposal integrates information about a $H\times W\times Z$ 3-D data in Fig. 1 into a quantum state having it formula in (2):

2 $$\begin{aligned} \left| D_3\right\rangle =\frac{1}{2^{\frac{h+w+l}{2}}}\left( \sum _{Y=0}^{H-1} \sum _{X=0}^{W-1}\sum _{Z=0}^{L-1} |S_{Y XZ}\rangle _{F}\otimes \left| Y X Z\right\rangle \right) , \end{aligned}$$

where

3 $$\begin{aligned} |Y X Z\rangle= & {} |Y\rangle |X\rangle |Z\rangle =\left| y_{0} y_{1} \ldots y_{h-1}\right\rangle \left| x_{0} x_{1} \ldots x_{w-1}\right\rangle |z_0z_1\ldots z_{l-1}\rangle ,~~ y_{i}, x_{i},z_{i} \in \{0,1\},\nonumber \\ |S_{YXZ}\rangle _F= & {} \left| s_{Y X Z}^{0}s_{Y X Z}^{1}\ldots s_{Y X Z}^{p}s_{Y X Z}^{p+1}\ldots s_{Y X Z}^{p+q-1}\right\rangle ,\quad s_{Y X Z}^{i}\in \{0,1\},\nonumber \\ h= & {} {\left\{ \begin{array}{ll} \left\lceil \log _{2} H\right\rceil , &{} H>1; \\ 1, &{} H=1, \end{array}\right. } ~ w= {\left\{ \begin{array}{ll} \left\lceil \log _{2}W\right\rceil ,&{} W>1;\\ 1,&{} W=1, \end{array}\right. } ~ l= {\left\{ \begin{array}{ll} \left\lceil \log _{2}L\right\rceil ,&{} L>1;\\ 1,&{} L=1. \end{array}\right. } \end{aligned}$$

Here, $|Y X Z\rangle$ is the location information, $|S_{YXZ}\rangle _F=|s_{YXZ}^{0}s_{YXZ}^{1}\ldots s_{YXZ}^{p}s_{YXZ}^{p+1}\ldots s_{YXZ}^{p+q-1}\rangle$ can store a floating-point number of 3-D data, and $\lceil {\cdot }\rceil$ denotes rounding up. Let $S_{YXZ}^{s}=s_{YXZ}^{0}$ is a sign qubit, an encoded exponent field of *p* qubits denoted by $S_{YXZ}^{e}=s_{YXZ}^{1}s_{YXZ}^{2}\ldots s_{YXZ}^{p}$ and a normalized binary fractional having $q-1$ qubits to the right of the radix point denoted by $S_{YXZ}^{f}=s_{YXZ}^{p+1}s_{YXZ}^{p+2}\ldots s_{YXZ}^{p+q-1}$. That is, floating-point format of $|S_{YXZ}\rangle _F$ is shown in Table 2, and each format’s parameters are shown in Table 3.

**Table 2:** Floating point format of $|S_{YXZ}\rangle _F$.

1	p	$q-1$
*s*	*e*	*f*
$s_{YXZ}^{0}$	$s_{YXZ}^{1}s_{YXZ}^{2}\ldots s_{YXZ}^{p}$	$s_{YXZ}^{p+1}s_{YXZ}^{p+2}\ldots s_{YXZ}^{p+q-1}$
$S_{YXZ}^{s}$	$S_{YXZ}^{e}$	$S_{YXZ}^{f}$

**Table 3:** Summary of format parameters.

Parameter	Format			
	Single	Single extended	Double	Double extended
p	8	$\ge 11$	11	$\ge 15$
q	24	$\ge 32$	53	$\ge 64$
Format width in bits	32	$\ge 43$	64	$\ge 79$

### Classical interpolation methods

**Figure 2:**
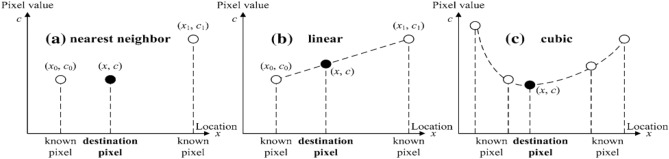
The principle of interpolation in one direction.

In image scaling, interpolation methods are necessary to produce new pixels (when scaling up) or delete redundant pixels (when scaling down). The commonly used interpolation methods include nearest neighbor, linear and cubic interpolation in Fig. 2:

As shown in Fig. 2, the value of the destination pixel (*x*, *c*) can be estimated by linear interpolation method, i.e.,

4 $$\begin{aligned} c=\frac{\left( x_{1}-x\right) c_{0}+\left( x-x_{0}\right) c_{1}}{x_{1}-x_{0}}. \end{aligned}$$

The bilinear interpolation method plays an important role in classical image scaling. For a $W\times H$ (width and height) image, the size of the corresponding interpolated image is $W^{\prime }\times H^{\prime }$, which can be described in two steps.

- *Step 1: Coordinate map.*

**Figure 3:**
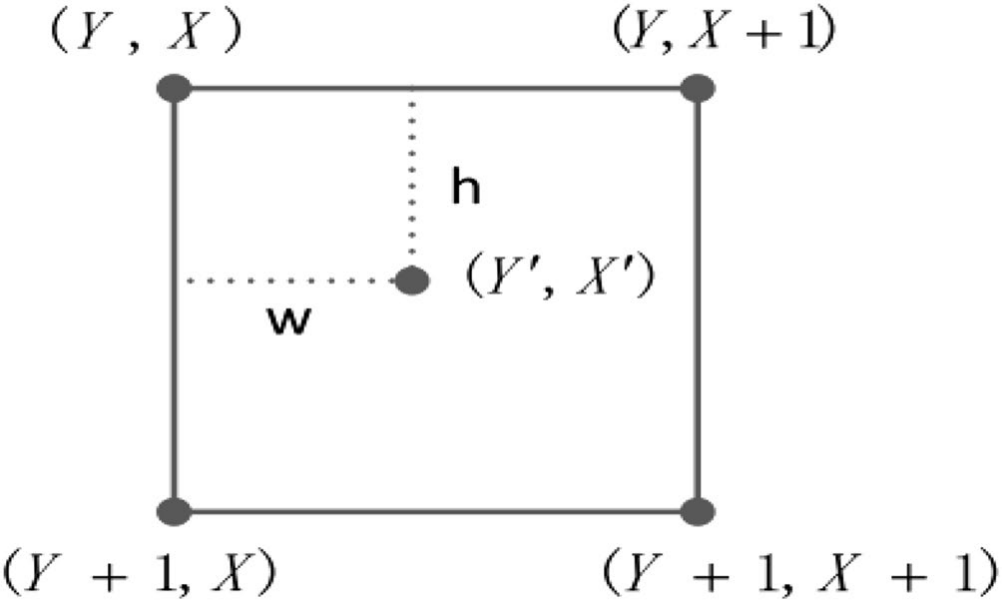
Coordinate map relationship.

The coordinate $(Y^{\prime }, X^{\prime })$ of the interpolated image is restored from the positions (*Y*, *X*), $(Y+1, X)$, $(Y, X+1)$ and $(Y+1, X+1)$ in the original image. The corresponding relationship is shown in Fig. 3. Here,

5 $$\begin{aligned} Y=\left\lfloor Y^{\prime } \times \frac{H}{H^{\prime }}\right\rfloor , ~~~X=\left\lfloor X^{\prime } \times \frac{W}{W^{\prime }}\right\rfloor , ~~~h=\frac{H}{H^{\prime }} Y^{\prime }-Y, ~~~w=\frac{W}{W^{\prime }} X^{\prime }-X, \end{aligned}$$

where $\lfloor {\cdot }\rfloor$ denotes rounding down.

- *Step 2: Calculating pixel value.*

The pixel value in position $(Y^{\prime }, X^{\prime })$ of the interpolated image using bilinear interpolation method can be calculated as follows:

6 $$\begin{aligned}&\left\{ \begin{aligned} f\left( Y^{\prime }, X^{\prime }\right) _{1}&=\left( 1-w\right) f(Y, X)+w f(Y, X+1) \\ f\left( Y^{\prime }, X^{\prime }\right) _{2}&=\left( 1-w\right) f(Y+1, X)+w f(Y+1, X+1) \end{aligned} \right. \nonumber \\&\quad \Rightarrow f\left( Y^{\prime }, X^{\prime }\right) =\left( 1-h\right) f\left( Y^{\prime }, X^{\prime }\right) _{1}+h f\left( Y^{\prime }, X^{\prime }\right) _{2} \nonumber \\&\quad\qquad\qquad\qquad =\left( 1-w\right) \left( 1-h\right) f(Y, X)+w\left( 1-h\right) f(Y, X+1)\nonumber \\&\qquad \qquad\qquad\qquad+\left( 1-w\right) h f(Y+1, X)+w h f(Y+1, X+1). \end{aligned}$$

Hance, the bilinear interpolation method is a single linear interpolation method in the *y* direction and two single linear interpolation methods in the *x* direction.

### Quantum Fourier transform

**Figure 4:**
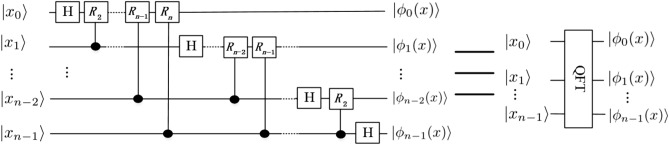
Quantum circuits that implement QFT. Not shown are swap gates at the end of the circuit which reverse the order of the qubits^31^.

The Quantum Fourier Transform, QFT, is an application of classical discrete Fourier transform to the quantum states. The QFT on an orthonormal basis $|0 \rangle , |1\rangle , \ldots , |N-1\rangle$ is defined to be a linear operator with the following action on the basis states:

7 $$\begin{aligned} QFT|j\rangle =\frac{1}{\sqrt{N}}\sum _{k=0}^{N-1}e^{\frac{2\pi ijk}{N}}|k\rangle , \end{aligned}$$

where *i* is an imaginary unit. The specific quantum circuits are shown in Fig. 4, where $|x\rangle =|x_0x_1\ldots x_{n-1}\rangle$. For simplicity, in this figure we have omitted the sequence of SWAP gates needed to invert the order of the output qubits.

We can equally define an Inverse Quantum Fourier Transform operator IQFT so that

8 $$\begin{aligned} IQFT|k\rangle =\frac{1}{\sqrt{N}}\sum _{j=0}^{N-1}e^{-\frac{2\pi ijk}{N}}|j\rangle . \end{aligned}$$

With the direct and the inverse Fourier transforms, we can move back and forth between the computational basis and the phase representation. In our notation, this conversion from the phase encoding to the computational basis $|x\rangle =|x_0x_1\ldots x_{n-1}\rangle$ is written as

$$\begin{aligned} IQFT\cdot QFT |x\rangle =|x\rangle . \end{aligned}$$

By employing 1-qubit Hadamard gates *H* and 2-qubit controlled-phase gates $CR_k$, the QFT and IQFT can be efficiently implemented.

## Quantum scaling up and down

In the following subsections, we first introduced some basic modules: *adding* *one*, *rotation*, *adder*, *multiplier*, *special* *subtractor* and *divided* *by* 2 modules, and then we designed the addition and multiplication of the floating-point based on QFT (*Q*-*Adder* and *Q*-*Multiplier*) and *Converter* for converting fixed-point numbers to floating-point numbers. Finally, we proposed the design scheme of quantum scaling up and down for 3-D floating-point data.

### Some modules

In the next subsection, a series of quantum modules are used. So we introduce their circuits in this subsection.

1. *Adding one module based on integer*

**Figure 5:**
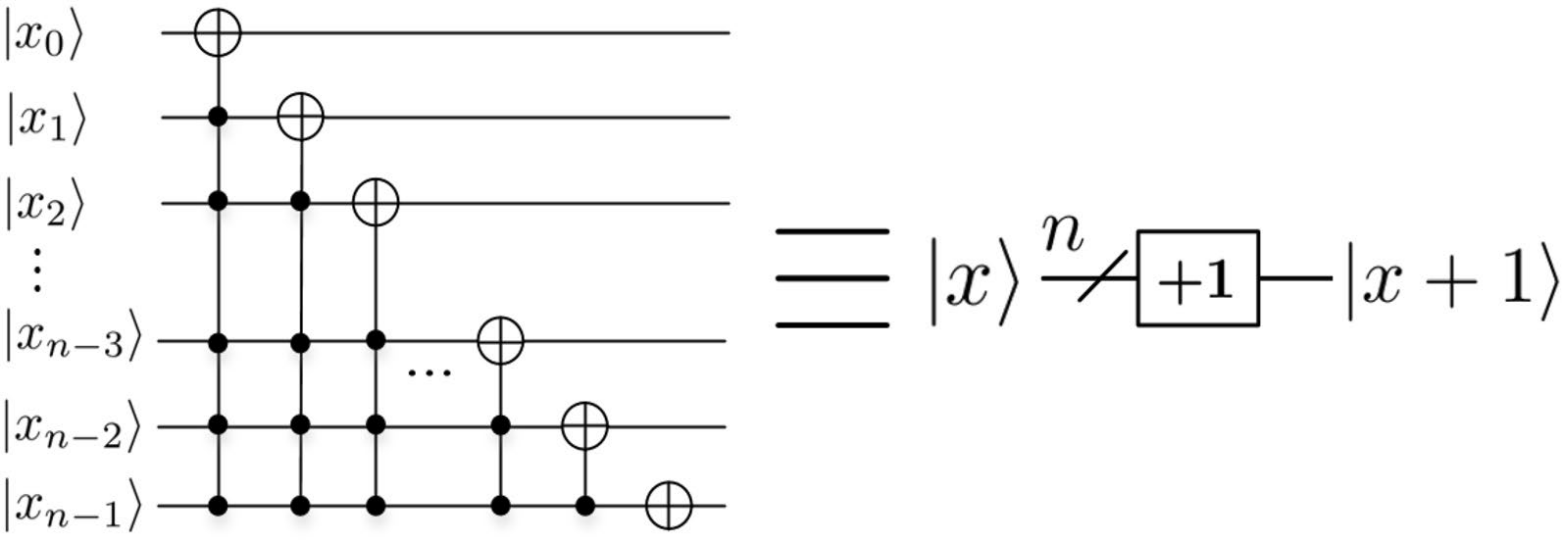
Adding one module^32^.

In this paper, we use the *adding* *one* module based on integer^32^, and its quantum circuit is shown in Fig. 5, where $|x\rangle =|x_0x_1\ldots x_{n-1}\rangle$, *n* is a positive integer, $n\ge 1$, $x_0, x_1, \ldots , x_{n-1}\in \{0, 1\}$.

2. *Rotation* *module*

**Figure 6:**
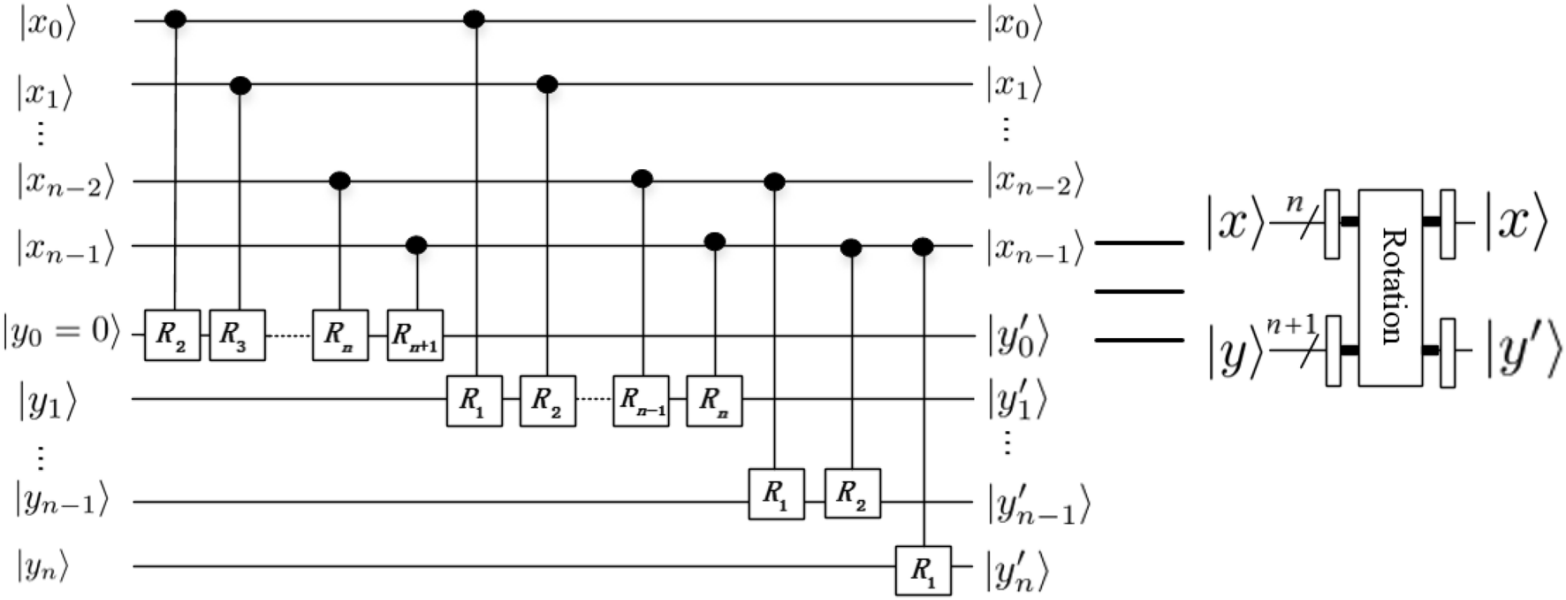
Rotation module^31^.

The operator of the *rotation* module can be expressed as

9 $$\begin{aligned} |x\rangle |y\rangle {\mathop {\longrightarrow }\limits ^{\text {Rotation}}}e^{i \frac{2\pi xy}{2^{ n+1}}}|x\rangle |y\rangle , \end{aligned}$$

and the corresponding quantum circuit is shown in Fig. 6, in other words, its effect is to introduce a phase shift in frequency domain, where $|x\rangle =|x_0x_1\ldots x_{n-1}\rangle$.

3. $$Adder \ and \ Multiplier \ modules$$

**Figure 7:**
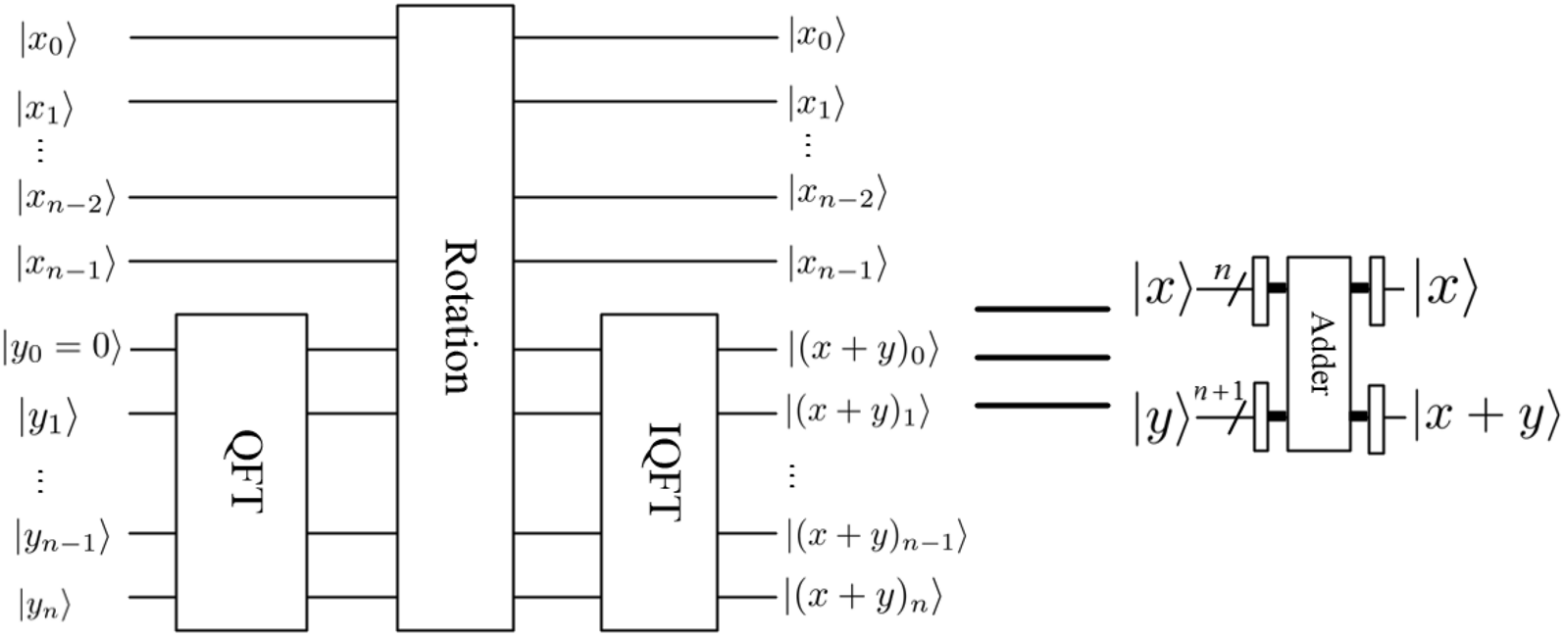
Adder module^29^.

**Figure 8:**
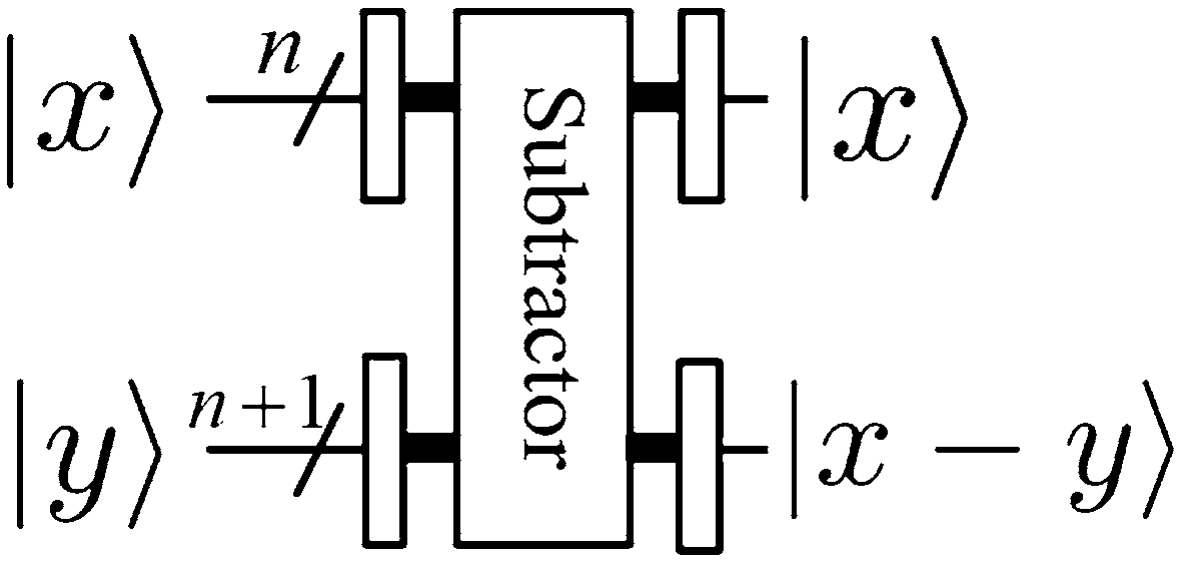
Subtractor module^29^.

**Figure 9:**
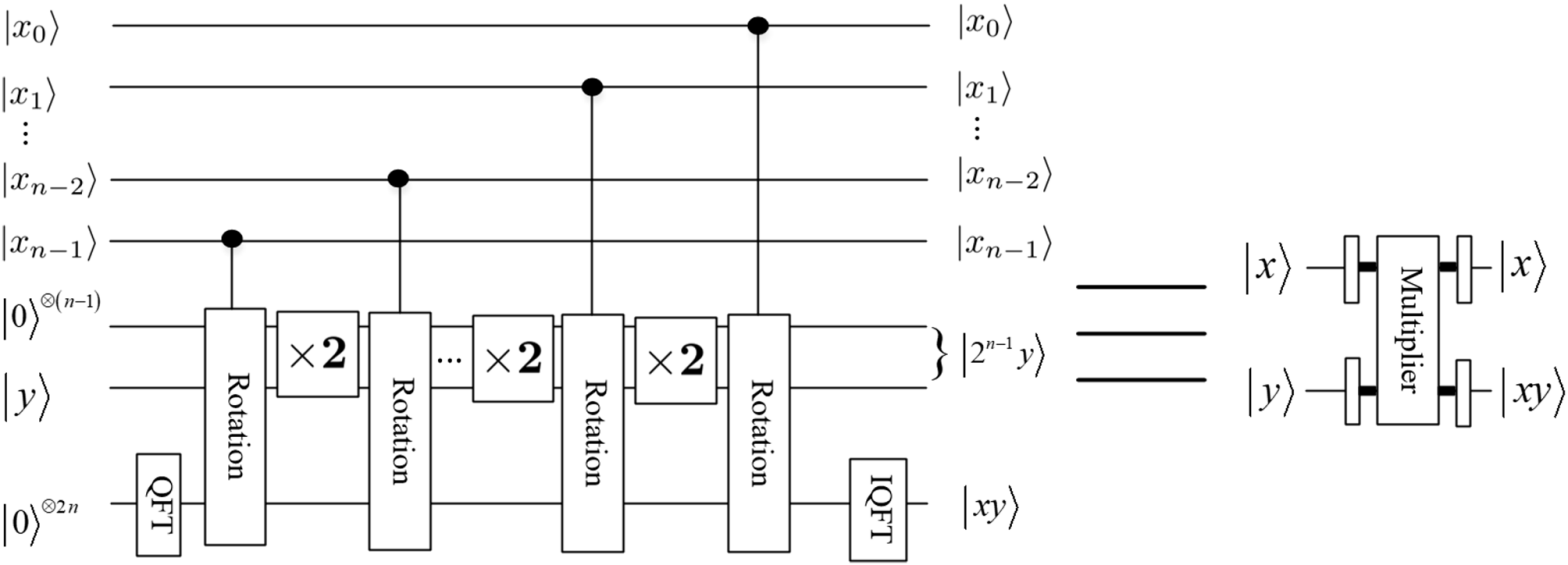
Multiplier module^29^.

Li^29^ proposed a new method for the design of two core modules (i.e., addition and multiplication) based on QFT. It is clear that the subtraction operation could be implemented by a bit of modification in $CR_k$. If we replace the element $e^{2\pi i/2^k}$ in $CR_k$ matrix with $e^{-2\pi i/2^k}$, then the quantum *adder* circuit would act as a quantum *subtractor* whose output will become $|x\rangle |x-y\rangle$. We don’t give the detail quantum circuit of the quantum *subtractor* module. The quantum circuits of quantum *adder* and *subtractor* and *multiplier* modules are shown in Figs 7,  8 and 9, respectively.

4. $$Special \ Subtractor \ module$$

**Figure 10:**
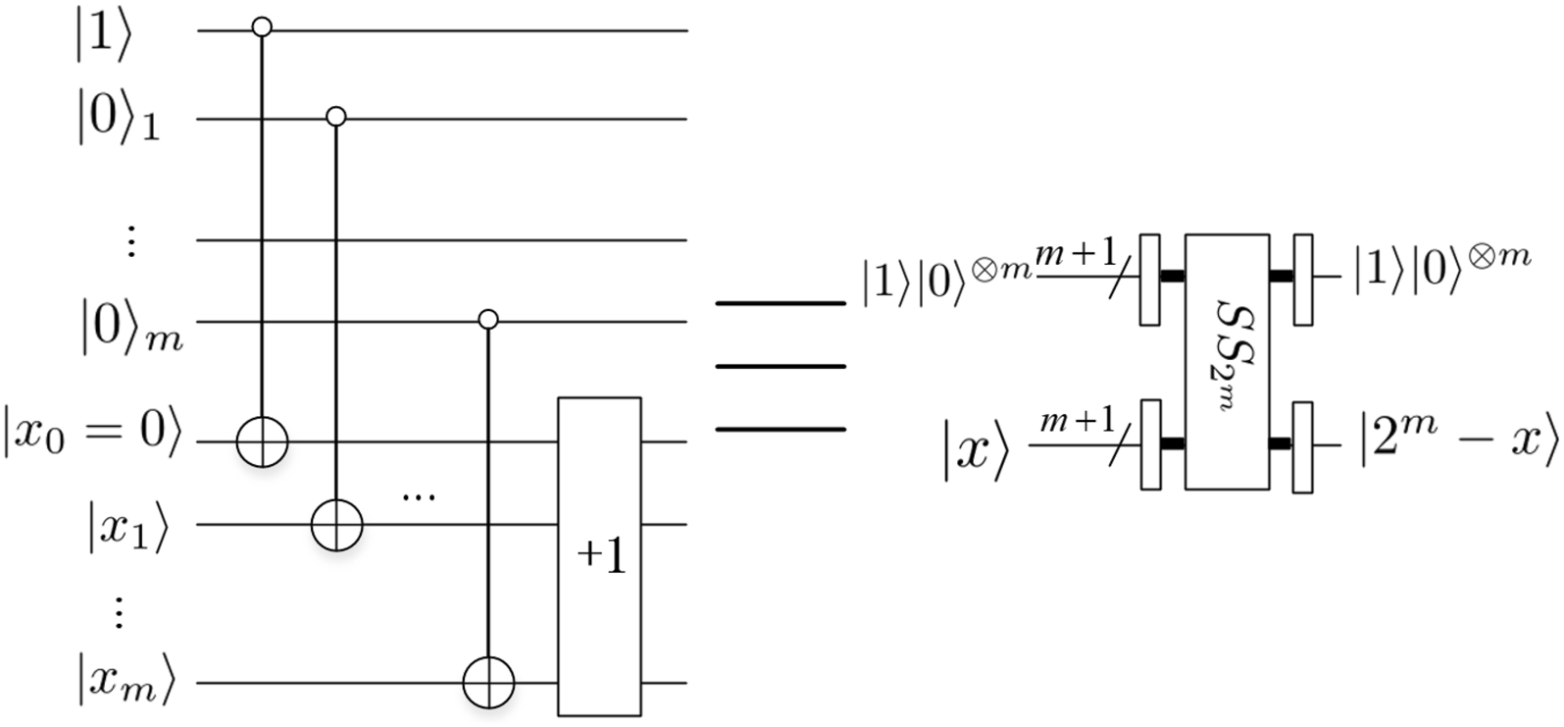
Special Subtractor module^29^.

The function of this module is to implement the subtraction of two *m*-qubit numbers, i.e. $2^{m}-x$, $0 \le x \le 2^{m}-1$, and the *special* *subtractor* quantum circuit is shown in Fig. 10.

5. $$Divided\ by \ 2$$ *module*

**Figure 11:**
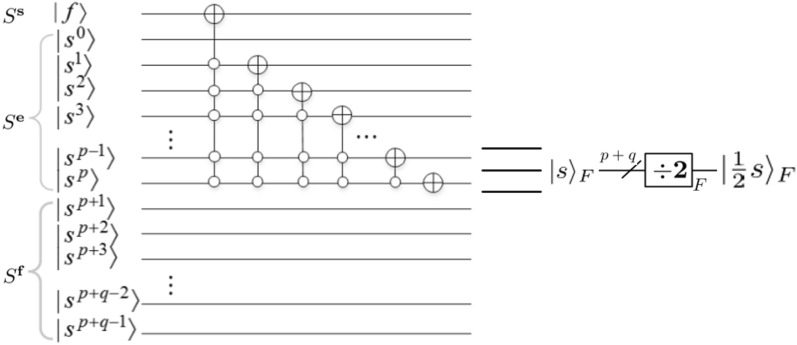
Divided by 2 module^12^.

Zhang et al.^12^ design the *divided* *by* 2 module in 2020. The *divided* *by* 2 module is to make a floating-point number $|s\rangle _F$ to $|\frac{1}{2}s\rangle _F$, where $|s\rangle _F=|s^0s^1\ldots s^ps^{p+1}\ldots s^{p+q-1}\rangle _F$. The circuit for floating-point *divided* *by* 2 module is depicted in Fig. 11.

### Floating-point addition and multiplication based on QFT

**Figure 12:**
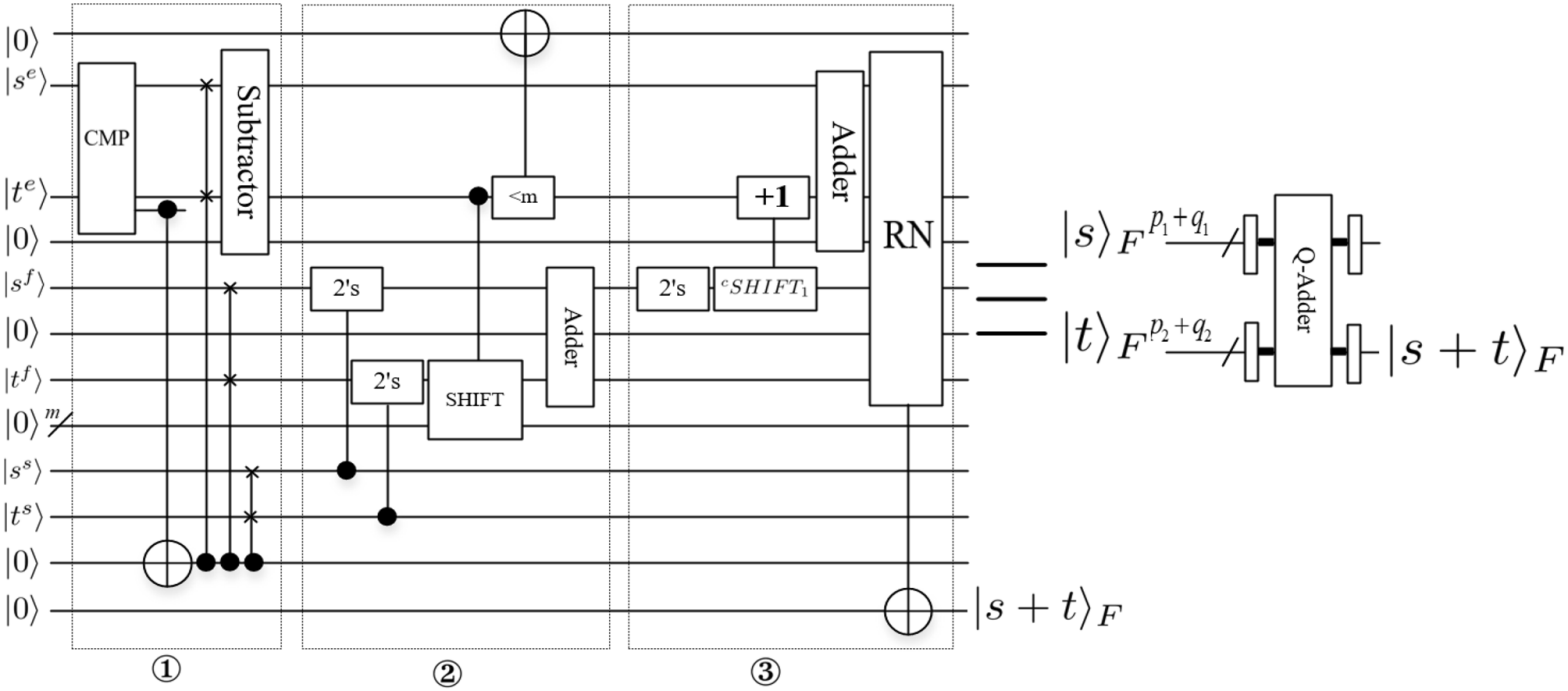
Q-Adder module.

**Figure 13:**
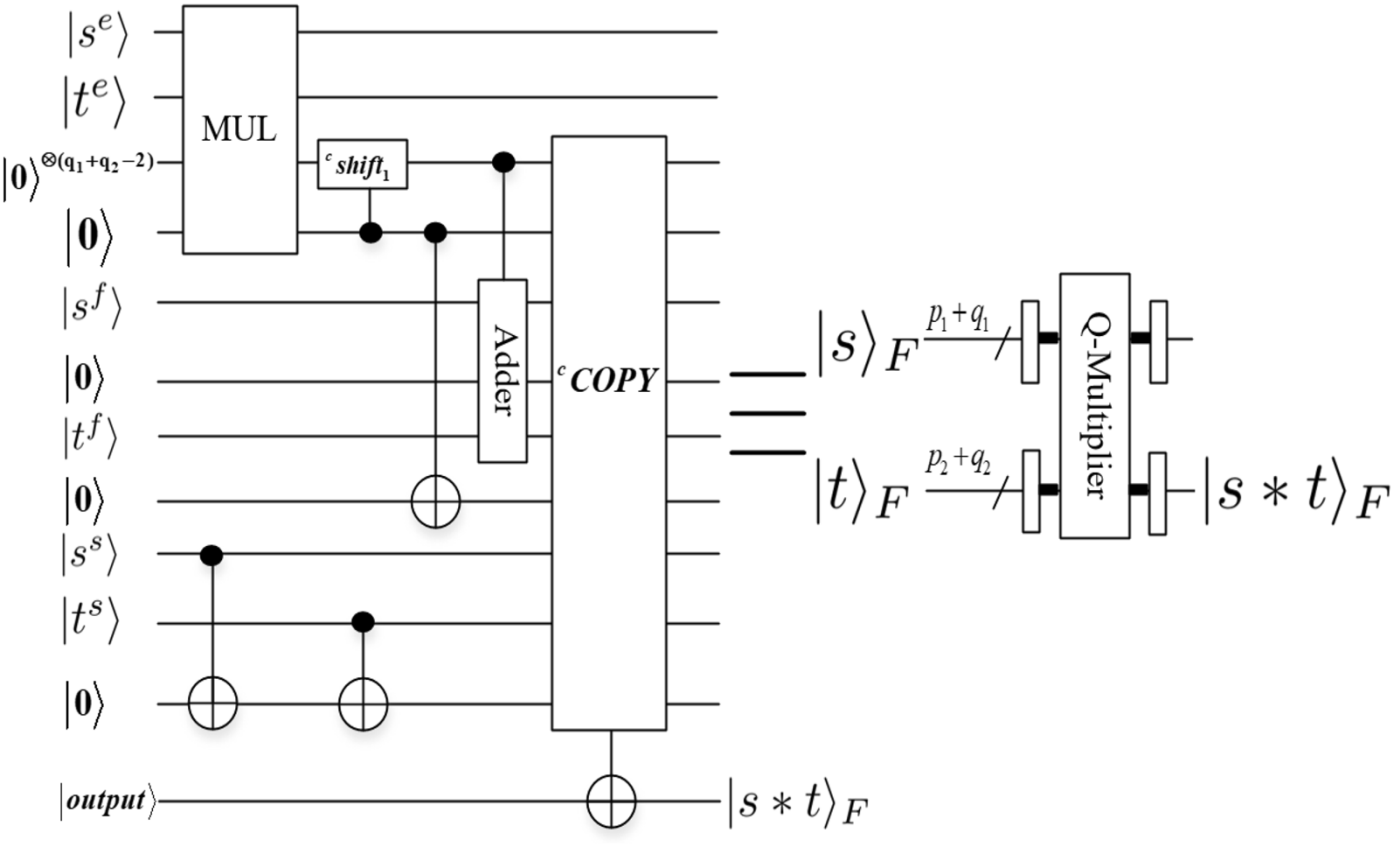
*Q*-Multiplier module.

**Figure 14:**
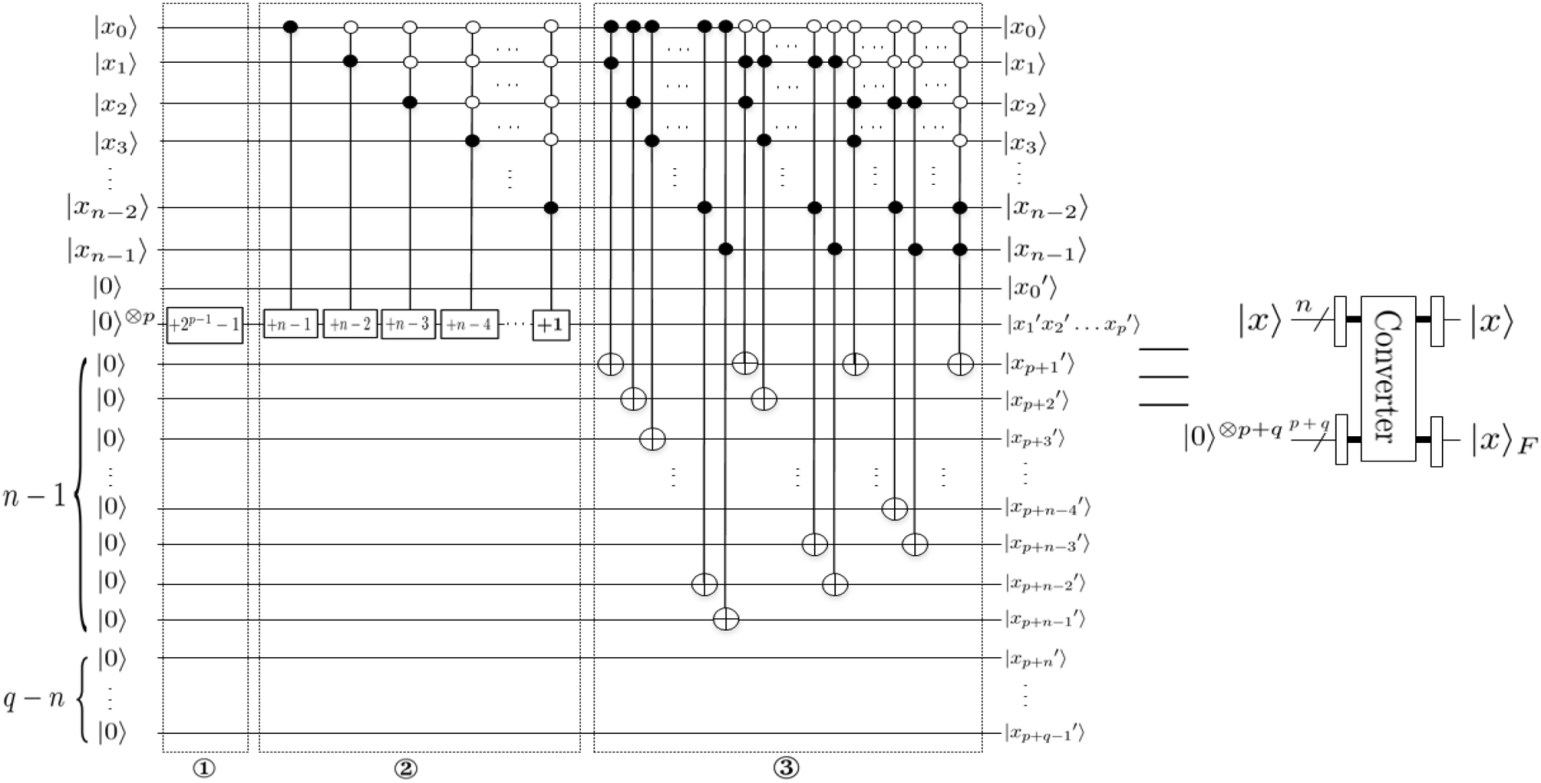
Converter module.

In the next subsection, the addition and multiplication of the floating-point based on QFT (*Q*-*Adder* and *Q*-*Multiplier*) and *Converter* module are designed. The QFT offers an interesting way to perform arithmetic operations on a quantum computer. Nielsen^31^ given the quantum circuit of the QFT. Adder and Multiplier modules^33^ based on floating-point numbers are given. We will benefit from the circuits^29,33^ in our quantum addition and multiplication circuits based on QFT. In this paper, we design the addition and multiplication based on QFT (*Q*-*Adder* and *Q*-*Multiplier*) operations, and the quantum circuits are depicted in Figs. 12 and 13. In order to have the same number of bits of the two floating-point numbers that are multiplied, we designed the *Converter* module for converting fixed-point numbers to floating-point numbers in the design scheme of quantum scaling for 3-D floating-point data, and the quantum circuit is depicted in Fig. 14. For convenience, other unremarked qubits are the garbage outputs.

1. *Q*-*Adder* *module*

Figure 12 shows the addition of two floating-point numbers $|s\rangle _F$ and $|t\rangle _F$, where $|s\rangle _F=|s^ss^es^f\rangle _F=|s^ss^1s^2\ldots s^{p_1}s^{p_1+1}\ldots s^{p_1+q_1-1}\rangle _F$, $|t\rangle _F$ $=|t^st^et^f\rangle _F$ $=|t^st^1t^2\ldots t^{p_2}$ $t^{p_2+1}\ldots$ $t^{p_2+q_2-1}\rangle _F$.

- Step ①. Determine result exponent $\bigtriangleup E=s^e-t^e$.

The comparator module based on integer (CMP) is used to compares two exponents information $s^e$ and $t^e$ of $|s\rangle _F$ and $|t\rangle _F$. If $s^e<t^e$ , swap the two floating-point numbers (comparison followed by controlled swaps). Align the two results according to the difference in exponents $\bigtriangleup E=s^e-t^e$. (only if $\bigtriangleup E<q_2-1$, else the adder will have no effect, $m=\{1,2,\ldots , q_2-1\}$ in ②).

- Step ②. Add mantissas in two’s complement.

Compute two’s complement from sign bits and mantissas (including the implicit leading 1). The second mantissa $|t^f\rangle$ is shifted by the difference of the exponents $s^e$ and $t^e$. Add mantissas in two’s complement.

- Step ③. Renormalize the intermediate result.

The final RN gate renormalizes the intermediate result using the first-ones circuit followed by shifting the mantissa by the output of the first-ones circuit (i.e., if adding the two mantissas in ② caused an overflow, right-shift the result by 1 and increment the exponent) and copies out the resulting floating-point representation.

2. *Q*-*Multiplier* module

Figure 13 shows the multiplication of two floating-point numbers $|s\rangle _F$ and $|t\rangle _F$, where $|s\rangle _F=|s^ss^es^f\rangle _F=|s^ss^1s^2\ldots s^{p_1}s^{p_1+1}\ldots s^{p_1+q_1-1}\rangle _F$, $|t\rangle _F=|t^st^et^f\rangle _F=|t^st^1t^2\ldots t^{p_2}t^{p_2+1}\ldots t^{p_2+q_2-1}\rangle _F$. There is only one renormalization step involved. In summary, it requires the following steps:

1. Determine result exponent.
2. Multiply mantissas (including the implicit leading 1) into a $(q_1+q_2-2)$-bit register.
3. If there was an overflow, right-shift by one and increment the result exponent.
4. The final step denoted by ${^c}COPY$ consists of conditionally copying out of the resulting exponent, mantissa and sign bit.

3. *Converter* *module*

In Fig. 14, *Converter* module converts fixed-point numbers to floating-point numbers, where $|x\rangle =|x_0x_1\ldots x_{n-1}\rangle$, $|x\rangle _F=|{x_0}^\prime {x_1}^\prime \ldots {x_p}^\prime {x_{p+1}}^\prime \ldots {x_{p+q-1}}^\prime \rangle _F$. Before explaining the circuit, we first need to normalize $|x\rangle$.

- Step ①. Calculate the bias value. That is, $+2^{p-1}-1$ module can be realized by *adding* *one* module $2^{p-1}-1$ times.
- Step ②. Calculate the value of the exponent code using the formula $exponent=bias~value+exponent~truth~value$, where the truth value of the exponent code is the value of exponent code after $|x\rangle$ normalization, $+n-1, +n-2, \ldots$, similar to Step ①.
- Step ③. Calculate the value of the mantissa. If $x_0=1, x_1=1$, the value of the mantissa can be realized using the Toffoli gate and zero padding at the end of $|x_1x_2\ldots x_{n-1}\rangle$. Therefore, the remaining cases (example $x_0=0, x_1=1$ and $x_0=0, x_1=0, x_2=1$) are carried out in this way in sequence. That is to say, determine the position of the first 1.

### Quantum scaling-up circuit for 3-D floating-point data

In this subsection, the quantum scaling-up circuit of 3-D floating-point data based on QFT using trilinear interpolation is designed. The key idea of the proposed circuit is mathematically explained in (13).

Without loss of generality, the quantum representation for an array of $2^h\times 2^w\times 2^l$ 3-D floating-point data in (2) ($H=2^h, W=2^w, L=2^l$) can be expressed as follows:

10 $$\begin{aligned} \left| D_3\right\rangle =\frac{1}{2^{\frac{h+w+l}{2}}}\left( \sum _{Y=0}^{2^h-1} \sum _{X=0}^{2^w-1}\sum _{Z=0}^{2^l-1} |S_{Y XZ}\rangle _{F}\otimes \left| Y X Z\right\rangle \right) , \end{aligned}$$

where

$$\begin{aligned} |Y X Z\rangle= & {} |Y\rangle |X\rangle |Z\rangle =\left| y_{0} y_{1} \ldots y_{h-1}\right\rangle \left| x_{0} x_{1} \ldots x_{w-1}\right\rangle |z_0z_1\ldots z_{l-1}\rangle ,~~ y_{i}, x_{i},z_{i} \in \{0,1\},\\ |S_{YXZ}\rangle _F= & {} \left| s_{Y X Z}^{0}s_{Y X Z}^{1}\ldots s_{Y X Z}^{p}s_{Y X Z}^{p+1}\ldots s_{Y X Z}^{p+q-1}\right\rangle ,\quad s_{Y X Z}^{i}\in \{0,1\}. \end{aligned}$$

The quantum representation uses *Y* (*h* *qubits*), *X* (*w* *qubits*), *Z* (*l* *qubits*), $|S_{YXZ}\rangle _F~(p+q~qubits)$, respectively, to denote the *Y*-coordinate, *X*-coordinate, *Z*-coordinate and a floating-point number of 3-D data.

Assume that there is an array of $2^h\times 2^w\times 2^l$ 3-D floating-point data and the size of the resulting data after being scaled is $2^{h+h_1}\times 2^{w+w_1}\times 2^{l+l_1}$, i.e., $r_y=2^{h_1}$, $r_x=2^{w_1}$ and $r_z=2^{l_1}$ (where $r_y$, $r_x$ and $r_z$ represent the scaling in *y*-coordinate, *x*-coordinate, *z*-coordinate axis directions, respectively). The trilinear interpolation method for 3-D floating-point data can be described within the following two steps in detail.

- *Step 1: Build coordinate map relationship.*

**Figure 15:**
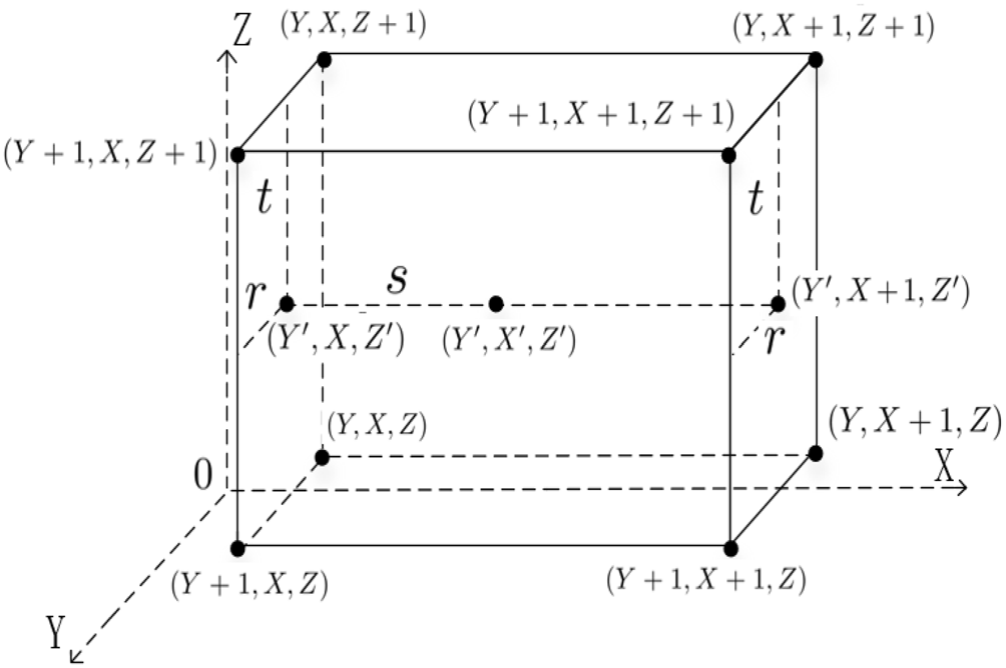
Coordinate map relationship for 3-D.

The coordinate position $(Y^{\prime }, X, Z^{\prime })$ of the interpolated data can build a map relationship with the four positions $(Y+1, X, Z+1)$, $(Y+1, X, Z)$, $(Y, X, Z+1)$ and (*Y*, *X*, *Z*) of the original data when *X* is fixed. The coordinate position $(Y^{\prime }, X+1, Z^{\prime })$ of the interpolated data can build a map relationship with the four positions $(Y+1, X+1, Z+1)$, $(Y+1, X+1, Z)$, $(Y, X+1, Z+1)$ and $(Y, X+1, Z)$ of the original data when $X+1$ is fixed. The coordinate position $(Y^{\prime }, X^{\prime }, Z^{\prime })$ of the interpolated data can build a map relationship with the two positions $(Y^{\prime }, X, Z^{\prime })$, $(Y^{\prime }, X+1, Z^{\prime })$ of the original data when *yoz* plane is fixed. The corresponding relationship is shown in Fig. 15. Therein,

11 $$\begin{aligned} Y=\left\lfloor \frac{Y^{\prime }}{2^{h_1}}\right\rfloor , X=\left\lfloor \frac{X^{\prime }}{2^{w_1}}\right\rfloor , ~~Z=\left\lfloor \frac{Z^{\prime }}{2^{l_1}}\right\rfloor , ~~r=\frac{Y^{\prime }}{2^{h_1}}-Y, ~~s=\frac{X^{\prime }}{2^{w_1}}-X, ~~t=\frac{Z^{\prime }}{2^{l_1}}-Z, \end{aligned}$$

where $\lfloor {\cdot }\rfloor$ represents the rounding down operation, $|Y^\prime \rangle =|Y_{h+h_1-1}Y_{h+h_1-2}\ldots Y_{1}Y_{0}\rangle$, $|X^\prime \rangle$ $=|X_{w+w_1-1}$ $X_{w+w_1-2}\ldots X_{1}X_{0}\rangle$, $|Z^\prime \rangle =|Z_{l+l_1-1}Z_{l+l_1-2}\ldots Z_{1}Z_{0}\rangle$. To build the mapping relationship described in Fig. 15, the multiply Control-Not operations and adding one operation $+1$ are chosen as the unitary operators. The function of the multiply Control-Not operators is to utilize *h* Control-Not gates to copy the *h* qubits $|Y_{h+h_1-1}Y_{h+h_1-2}\ldots Y_{h_1}\rangle$ into the *h* ancillary qubits $|0\rangle ^{\otimes h}$. Through these two unitary operators, the interpolation mapping relationship between the position of original data and the interpolated data has been established. The details are described in Figs. 17 and 18.

- *Step 2: Calculating value for 3-D floating-point data.*

According to (6), the value for 3-D floating-point data in position $(Y^{\prime }, X^{\prime }, Z^{\prime })$ of the interpolated data using trilinear interpolation can be calculated as follows:

12 $$\begin{aligned} |S_{Y^{\prime }, X, Z^{\prime }}\rangle&=(1-t)|S_{Y^{\prime }, X, Z^{\prime }}\rangle _1 +t|S_{Y^{\prime }, X, Z^{\prime }}\rangle _2\nonumber \\&= (1-r)(1-t)|S_{Y+1, X, Z+1}\rangle +r(1-t) |S_{Y, X, Z+1}\rangle \nonumber \\&\quad +(1-r)t|S_{Y+1, X, Z}\rangle +rt |S_{Y, X, Z}\rangle .\nonumber \\ |S_{Y^{\prime }, X+1, Z^{\prime }}\rangle&=(1-t)|S_{Y^{\prime }, X+1, Z^{\prime }}\rangle _1 +t|S_{Y^{\prime }, X+1, Z^{\prime }}\rangle _2\nonumber \\&= (1-r)(1-t)|S_{Y+1, X+1, Z+1}\rangle +r(1-t) |S_{Y, X+1, Z+1}\rangle \nonumber \\&\quad +(1-r)t|S_{Y+1, X+1, Z}\rangle +rt |S_{Y, X+1, Z}\rangle .\nonumber \\ \Rightarrow |S_{Y^{\prime }, X^{\prime }, Z^{\prime }}\rangle&=(1-s)|S_{Y^{\prime }, X, Z^{\prime }}\rangle +s|S_{Y^{\prime }, X+1, Z^{\prime }}\rangle \nonumber \\&=(1-r)(1-t)(1-s)|S_{Y+1, X, Z+1}\rangle +r(1-t)(1-s)|S_{Y, X, Z+1}\rangle \nonumber \\&\quad +(1-r)t(1-s)|S_{Y+1, X, Z}\rangle +rt(1-s) |S_{Y, X, Z}\rangle \nonumber \\&\quad + (1-r)(1-t)s|S_{Y+1, X+1, Z+1}\rangle +r(1-t)s |S_{Y, X+1, Z+1}\rangle \nonumber \\&\quad +(1-r)ts|S_{Y+1, X+1, Z}\rangle +rts |S_{Y, X+1, Z}\rangle . \end{aligned}$$

Therefore,

13 $$\begin{aligned} |S_{Y^{\prime }, X^{\prime }, Z^{\prime }}\rangle&=\left[ 1-\left( \frac{Y^{\prime }}{2^{h_1}}-Y\right) \right] \left[ 1-\left( \frac{Z^{\prime }}{2^{l_1}}-Z\right) \right] \left[ 1-\left( \frac{X^{\prime }}{2^{w_1}}-X\right) \right] |S_{Y+1, X, Z+1}\rangle \nonumber \\&\quad +\left( \frac{Y^{\prime }}{2^{h_1}}-Y\right) \left[ 1-\left( \frac{Z^{\prime }}{2^{l_1}}-Z\right) \right] \left[ 1-\left( \frac{X^{\prime }}{2^{w_1}}-X\right) \right] |S_{Y, X, Z+1}\rangle \nonumber \\&\quad +\left[ 1-\left( \frac{Y^{\prime }}{2^{h_1}}-Y\right) \right] \left( \frac{Z^{\prime }}{2^{l_1}}-Z\right) \left[ 1-\left( \frac{X^{\prime }}{2^{w_1}}-X\right) \right] |S_{Y+1, X, Z}\rangle \nonumber \\&\quad +\left( \frac{Y^{\prime }}{2^{h_1}}-Y\right) \left( \frac{Z^{\prime }}{2^{l_1}}-Z\right) \left[ 1-\left( \frac{X^{\prime }}{2^{w_1}}-X\right) \right] |S_{Y, X, Z}\rangle \nonumber \\&\quad + \left[ 1-\left( \frac{Y^{\prime }}{2^{h_1}}-Y\right) \right] \left[ 1-\left( \frac{Z^{\prime }}{2^{l_1}}-Z\right) \right] \left( \frac{X^{\prime }}{2^{w_1}}-X\right) |S_{Y+1, X+1, Z+1}\rangle \nonumber \\&\quad +\left( \frac{Y^{\prime }}{2^{h_1}}-Y\right) \left[ 1-\left( \frac{Z^{\prime }}{2^{l_1}}-Z\right) \right] \left( \frac{X^{\prime }}{2^{w_1}}-X\right) |S_{Y, X+1, Z+1}\rangle \nonumber \\&\quad +\left[ 1-\left( \frac{Y^{\prime }}{2^{h_1}}-Y\right) \right] \left( \frac{Z^{\prime }}{2^{l_1}}-Z\right) \left( \frac{X^{\prime }}{2^{w_1}}-X\right) |S_{Y+1, X+1, Z}\rangle \nonumber \\&\quad +\left( \frac{Y^{\prime }}{2^{h_1}}-Y\right) \left( \frac{Z^{\prime }}{2^{l_1}}-Z\right) \left( \frac{X^{\prime }}{2^{w_1}}-X\right) |S_{Y, X+1, Z}\rangle . \nonumber \\&=\bigg \{[2^{h_1}-(Y^{\prime }-2^{h_1}Y)] [2^{l_1}-(Z^{\prime }-2^{l_1}Z)] [2^{w_1}-(X^{\prime }-2^{w_1}X)] |S_{Y+1, X, Z+1}\rangle \nonumber \\&\quad +(Y^{\prime }-2^{h_1}Y) [2^{l_1}-(Z^{\prime }-2^{l_1}Z)] [2^{w_1}-(X^{\prime }-2^{w_1}X)] |S_{Y, X, Z+1}\rangle \nonumber \\&\quad +[2^{h_1}-(Y^{\prime }-2^{h_1}Y)] (Z^{\prime }-2^{l_1}Z) [2^{w_1}-(X^{\prime }-2^{w_1}X)] |S_{Y+1, X, Z}\rangle \nonumber \\&\quad +(Y^{\prime }-2^{h_1}Y) (Z^{\prime }-2^{l_1}Z) [2^{w_1}-(X^{\prime }-2^{w_1}X)] |S_{Y, X, Z}\rangle \nonumber \\&\quad + [2^{h_1}-(Y^{\prime }-2^{h_1}Y)] [2^{l_1}-(Z^{\prime }-2^{l_1}Z)] (X^{\prime }-2^{w_1}X) |S_{Y+1, X+1, Z+1}\rangle \nonumber \\&\quad +(Y^{\prime }-2^{h_1}Y) [2^{l_1}-(Z^{\prime }-2^{l_1}Z)] (X^{\prime }-2^{w_1}X) |S_{Y, X+1, Z+1}\rangle \nonumber \\&\quad +[2^{h_1}-(Y^{\prime }-2^{h_1}Y)] (Z^{\prime }-2^{l_1}Z) (X^{\prime }-2^{w_1}X) |S_{Y+1, X+1, Z}\rangle \nonumber \\&\quad +(Y^{\prime }-2^{h_1}Y) (Z^{\prime }-2^{l_1}Z) (X^{\prime }-2^{w_1}X) |S_{Y, X+1, Z}\rangle \bigg \}\div 2^{h_1}\div 2^{w_1}\div 2^{l_1}. \end{aligned}$$

According to (11), $(Y^\prime -2^{h_1}Y)$ and $(X^\prime -2^{w_1}X)$ and $(Z^\prime -2^{l_1}Z)$ in (13) are the remainder of $Y^\prime /2^{h_1}$ and $X^\prime /2^{w_1}$ and $Z^\prime /2^{l_1}$, respectively. From (13), in order to prepare the floating-point data $|S_{Y^{\prime }, X^{\prime }, Z^{\prime }}\rangle$ in position $(Y^{\prime }, X^{\prime }, Z^{\prime })$ of the resulting data, the floating-point data $|S_{Y+1, X, Z+1}\rangle$, $|S_{Y+1, X, Z}\rangle$, $|S_{Y, X, Z+1}\rangle$, $|S_{Y, X, Z}\rangle$, $|S_{Y+1, X+1, Z+1}\rangle$, $|S_{Y+1, X+1, Z}\rangle$, $|S_{Y, X+1, Z+1}\rangle$ and $|S_{Y, X+1, Z}\rangle$ in positions $(Y+1, X, Z+1)$, $(Y+1, X, Z)$, $(Y, X, Z+1)$, (*Y*, *X*, *Z*), $(Y+1, X+1, Z+1)$, $(Y+1, X+1, Z)$, $(Y, X+1, Z+1)$ and $(Y, X+1, Z)$ of the original data need to be prepared first. Trilinear interpolation method utilizes these eight different positions of the original position (*Y*, *X*, *Z*) to map into one position $(Y^{\prime }, X^{\prime }, Z^{\prime })$ of the resulting data as shown in Fig. 15. The size of an array of given original data is known. At the same time, the size of the resulting data is known under a certain scaling ratio. Therefore, $(Y^{\prime }, X^{\prime }, Z^{\prime })$ can be considered as the input state when designing quantum circuit. Figs. 17 and 18 provide scaling-up circuit of 3-D floating-point data based on QFT using trilinear interpolation method.

Next, we explain the workflow of this circuit.

The inputs of this circuit are eight identical original data (denoted by $|S_{Y+1, X, Z+1}\rangle$, $|S_{Y+1, X, Z}\rangle$, $|S_{Y, X, Z+1}\rangle$, $|S_{Y, X, Z}\rangle$, $|S_{Y+1, X+1, Z+1}\rangle$, $|S_{Y+1, X+1, Z}\rangle$, $|S_{Y, X+1, Z+1}\rangle$ and $|S_{Y, X+1, Z}\rangle$) and the data positions in the scaled-up data denoted by $(Y+1, X, Z+1)$, $(Y+1, X, Z)$, $(Y, X, Z+1)$, (*Y*, *X*, *Z*), $(Y+1, X+1, Z+1)$, $(Y+1, X+1, Z)$, $(Y, X+1, Z+1)$ and $(Y, X+1, Z)$), where the subscript indicates the number of qubits. The output of this circuit is the scaled-up data denoted by $|S_{Y^{\prime }, X^{\prime }, Z^{\prime }}\rangle$.

Firstly, eight quantum oracle operators $\Omega _{Y+1, X, Z+1}$, $\Omega _{Y+1, X, Z}$, $\Omega _{Y, X, Z+1}$, $\Omega _{Y, X, Z}$, $\Omega _{Y+1, X+1, Z+1}$, $\Omega _{Y+1, X+1, Z}$, $\Omega _{Y, X+1, Z+1}$ and $\Omega _{Y, X+1, Z}$ are used to compute the original data values of $|S_{Y+1, X, Z+1}\rangle$, $|S_{Y+1, X, Z}\rangle$, $|S_{Y, X, Z+1}\rangle$, $|S_{Y, X, Z}\rangle$, $|S_{Y+1, X+1, Z+1}\rangle$, $|S_{Y+1, X+1, Z}\rangle$, $|S_{Y, X+1, Z+1}\rangle$ and $|S_{Y, X+1, Z}\rangle$, respectively. A quantum oracle operator $\Omega _{Y, X, Z}$ can realize the aim of assigning floating-point data $|S_{Y, X, Z}\rangle$ to the ancillary qubits $|0\rangle ^{\otimes (p+q)}$, which can be expressed by (14)

14 $$\begin{aligned} \Omega _{Y, X, Z}=\bigotimes \limits _{i=0}^{p+q-1}\Omega _{Y, X, Z}^{i}, \end{aligned}$$

the function of $\Omega _{Y, X, Z}^{i}$ is setting the value of the *i*th qubit of the data in (Y,X,Z):

15 $$\begin{aligned} \Omega _{Y, X, Z}^{i} :|0\rangle \rightarrow \left| 0 \oplus s_{Y, X, Z}^{i}\right\rangle , \end{aligned}$$

where $\bigoplus$ is the XOR operation, $s_{Y, X, Z}^{i}\in \{0, 1\}$. $\Omega _{Y, X, Z}$ are used to perform ($p+q$) XOR gate on the ($p+q$) $|0\rangle$ to obtain the data, as shown in following equation:

16 $$\begin{aligned} \Omega _{Y,X,Z}|0\rangle ^{\otimes (p+q)}=\bigotimes _{i=0}^{p+q-1} \left( \Omega _{Y,X,Z}^{i}|0\rangle \right) =\bigotimes _{i=0}^{p+q-1} \left| 0 \oplus s_{Y,X,Z}^{i}\right\rangle =\bigotimes _{i=0}^{p+q-1} \left| s_{Y,X,Z}^{i}\right\rangle =\left| S_{Y,X,Z}\right\rangle _F. \end{aligned}$$

Then, three *Special* *Subtraction* modules are used to obtain $2^{w_1}-x^{\prime }$, $2^{h_1}-y^{\prime }$, $2^{l_1}-z^{\prime }$, where $x^{\prime }=X^{\prime }-2^{w_1}X$, $y^{\prime }=Y^{\prime }-2^{h_1}Y$, $z^{\prime }=Z^{\prime }-2^{l_1}Z$, respectively. Further, eight groups of intermediate results $(2^{h_1}-y^{\prime })(2^{l_1}-z^{\prime })(2^{w_1}-x^{\prime })|S_{Y+1, X, Z+1}\rangle$, $y^{\prime }(2^{l_1}-z^{\prime })(2^{w_1}-x^{\prime })|S_{Y, X, Z+1}\rangle$, $z^{\prime }(2^{h_1}-y^{\prime })(2^{w_1}-x^{\prime })|S_{Y+1, X, Z}\rangle$, $y^{\prime }z^{\prime }(2^{w_1}-x^{\prime })|S_{Y, X, Z}\rangle$, $x^{\prime }(2^{h_1}-y^{\prime })(2^{l_1}-z^{\prime })|S_{Y+1, X+1, Z+1}\rangle$, $y^{\prime }x^{\prime }(2^{l_1}-z^{\prime })|S_{Y, X+1, Z+1}\rangle$, $z^{\prime }x^{\prime }(2^{h_1}-y^{\prime })|S_{Y+1, X+1, Z}\rangle$, $y^{\prime }z^{\prime }x^{\prime }|S_{Y, X+1, Z}\rangle$ are obtained. Here, we need to point out that the multiplication between the first three items in each group is the fixed-point multiplication based on QFT (*Multiplier* module, given by Fig. 9). Since the *Multiplier* module requires the qubits to be the same, we obtain the final number of qubits by seeking the maximum value. The number of qubits after multiplying the first three items in each group is $n_1=\max \{h_1, w_1+1, l_1\}$, $n_2=\max \{h_1+1, w_1+1, l_1\}$, $n_3=\max \{h_1, w_1+1, l_1+1\}$, $n_4=\max \{h_1+1, w_1+1, l_1+1\}$, $n_5=\max \{h_1, w_1, l_1\}$, $n_6=\max \{h_1+1, w_1, l_1\}$, $n_7=\max \{h_1, w_1, l_1+1\}$, $n_8=\max \{h_1+1, w_1, l_1+1\}$, respectively. Before multiplying with the fourth item floating-point data, the result of the previous fixed-point multiplication needs to be converted into the floating-point data of the same qubit through the *Converter* module, given by Fig. 14. Finally, the multiplication here uses the floating-point multiplier based on QFT (*Q*-*Multiplier* module, given by Fig. 13). For convenience, we omit the process of seeking the maximum value in the *Multiplier* module and the *Converter* module before the *Q*-*Multiplier* module, refer to Figs. 17 and 18.

Finally, 3-D floating-point data $|S_{Y^{\prime }, X^{\prime }, Z^{\prime }}\rangle$ of the scaled-up is obtained by using seven *Q*-*Adder* modules (given by Fig. 12) and $h_1+w_1+l_1$ *Divided* *by* 2 modules (given by Fig. 11) . So far, we have completed the image scaling-up operation based on trilinear interpolation method. The scaling-up circuit of 3-D floating-point data is given by Figs. 17 and 18. For convenience, other unremarked qubits are the garbage outputs. Therefore, Figs. 17 and 18 are simplified scaling-up circuit, where ancillary and garbage outputs are omitted.

**Figure 16:**
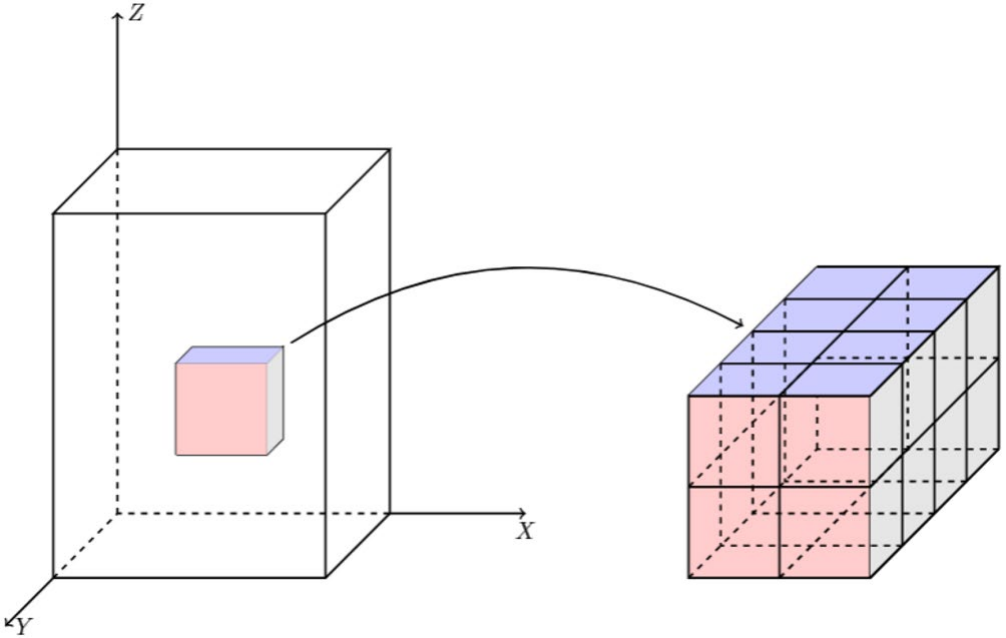
Schematic representation of the scaling-up circuit, where $r_y=4$, $r_x=2$, $r_z=2$.

For convenience, we show the schematic representation of the scaling-up circuit as Fig. 16, where the small cube represents a voxel point of the 3-D floating-point data, the right side is the scaled-up data representation, $r_y=4$, $r_x=2$, $r_z=2$.

### Quantum scaling-down circuit for 3-D floating-point data

Assume that there is an array of $2^{h+h_1}\times 2^{w+w_1}\times 2^{l+l_1}$ 3-D floating-point data and the size of the resulting data after being scaled is $2^{h}\times 2^{w}\times 2^{l}$, i.e., $r_y=2^{-h_1}$, $r_x=2^{-w_1}$ and $r_z=2^{-l_1}$ (where $r_y$, $r_x$ and $r_z$ represent the scaling in *y*-coordinate, *x*-coordinate, *z*-coordinate axis directions, respectively). Therefore, the scaling-down value for 3-D floating-point data in position $(Y^{\prime }, X^{\prime }, Z^{\prime })$ of the interpolated data using trilinear interpolation can be calculated as follows:

17 $$\begin{aligned} |S_{Y^{\prime }, X^{\prime }, Z^{\prime }}\rangle&=\bigg \{[2^{h_1}-(Y-2^{h_1}Y^{\prime })] [2^{l_1}-(Z-2^{l_1}Z^{\prime })] [2^{w_1}-(X-2^{w_1}X^{\prime })] |S_{Y+1, X, Z+1}\rangle \nonumber \\&\quad +(Y-2^{h_1}Y^{\prime }) [2^{l_1}-(Z-2^{l_1}Z^{\prime })] [2^{w_1}-(X-2^{w_1}X^{\prime })] |S_{Y, X, Z+1}\rangle \nonumber \\&\quad +[2^{h_1}-(Y-2^{h_1}Y^{\prime })] (Z-2^{l_1}Z^{\prime }) [2^{w_1}-(X-2^{w_1}X^{\prime })] |S_{Y+1, X, Z}\rangle \nonumber \\&\quad +(Y-2^{h_1}Y^{\prime }) (Z-2^{l_1}Z^{\prime }) [2^{w_1}-(X-2^{w_1}X^{\prime })] |S_{Y, X, Z}\rangle \nonumber \\&\quad + [2^{h_1}-(Y-2^{h_1}Y^{\prime })] [2^{l_1}-(Z-2^{l_1}Z^{\prime })] (X-2^{w_1}X^{\prime }) |S_{Y+1, X+1, Z+1}\rangle \nonumber \\&\quad +(Y-2^{h_1}Y^{\prime }) [2^{l_1}-(Z-2^{l_1}Z^{\prime })] (X-2^{w_1}X^{\prime }) |S_{Y, X+1, Z+1}\rangle \nonumber \\&\quad +[2^{h_1}-(Y-2^{h_1}Y^{\prime })] (Z-2^{l_1}Z^{\prime }) (X-2^{w_1}X^{\prime }) |S_{Y+1, X+1, Z}\rangle \nonumber \\&\quad +(Y-2^{h_1}Y^{\prime }) (Z-2^{l_1}Z^{\prime }) (X-2^{w_1}X^{\prime }) |S_{Y, X+1, Z}\rangle \bigg \}\div 2^{h_1}\div 2^{w_1}\div 2^{l_1}. \end{aligned}$$

Figures 19 and 20 provide scaling-down circuit of 3-D floating-point data based on QFT using trilinear interpolation method.

Next, we explain the workflow of this circuit.

Firstly, eight quantum oracle operators $\Omega _{Y+1, X, Z+1}$, $\Omega _{Y+1, X, Z}$, $\Omega _{Y, X, Z+1}$, $\Omega _{Y, X, Z}$, $\Omega _{Y+1, X+1, Z+1}$, $\Omega _{Y+1, X+1, Z}$, $\Omega _{Y, X+1, Z+1}$ and $\Omega _{Y, X+1, Z}$ are used to compute the original data values of $|S_{Y+1, X, Z+1}\rangle$, $|S_{Y+1, X, Z}\rangle$, $|S_{Y, X, Z+1}\rangle$, $|S_{Y, X, Z}\rangle$, $|S_{Y+1, X+1, Z+1}\rangle$, $|S_{Y+1, X+1, Z}\rangle$, $|S_{Y, X+1, Z+1}\rangle$ and $|S_{Y, X+1, Z}\rangle$, respectively.

Then, *adding* *one*, *Special* *Subtraction*, *Multiplier*, *Converter* and *Q*-*Multiplier* modules are used to design the scaling-down circuit of 3-D floating-point data.

Finally, 3-D floating-point data $|S_{Y^{\prime }, X^{\prime }, Z^{\prime }}\rangle$ of the scaled-down is obtained using seven *Q*-*Adder* modules and $(h_1+w_1+l_1)$ *Divided* *by* 2 modules. So far, we have completed the image scaling-down operation using trilinear interpolation method. For convenience, other unremarked qubits are the garbage outputs. Therefore, Figs. 19 and 20 are simplified scaling-down circuit, where ancillary and garbage outputs are omitted.

### Complexity analysis

The circuit network complexity depends on the number of elementary gate in quantum image processing (QIMP). The complexity of the basic quantum gate is considered to be one, including NOT gate, Control-Not gate and any $2\times 2$ unitary operator^31^.

The network complexities of *adding* *one*, *QFT*, *IQFT*, *rotation*, *adder* and *subtractor* modules are all $O(n^2)$^29,31^. The *Multiplier* module consists of 1 *QFT*, $(n-1)$ *Multiply* *by* 2, *n* *Rotation*, and 1 *IQFT*, and so, the complexity of *Multiplier* module is $O(n^3)$^29^. For the *Special* *Subtractor* module, Fig. 10 shows that it consists of $(m+1)$ CNOT gates and 1 *adding* *one* module, and so, the complexity of *Special* *Subtractor* module is $O(m^{2})$. An *m*-controlled NOT gate in the *divided* *by* 2 module can be decomposed into $2(m-1)$ Toffoli gates and 1 CNOT gate and the Toffoli gate can be approximately simulated by 6 CNOT gates^31^, so the complexity of it is $O(12m-11)$. Hence, the complexity of preparing the *divided* *by* 2 module is $O(p^2)$. In (16), if $s_{Y, X, Z}^{i}=1, \Omega _{Y, X, Z}^{i}$ is a $(h+w+l)$-controlled NOT qubit gate. Otherwise, it is a quantum identity gate. That is to say, every oracle operator $\Omega _{Y, X, Z}^{i}$, $i=0, \ldots , p+q-1$, is at most a $(h+w+l)$-controlled NOT qubit gate. For other oracle operators $\Omega _{Y+1, X, Z+1}$, $\Omega _{Y+1, X, Z}$, $\Omega _{Y, X, Z+1}$, $\Omega _{Y+1, X+1, Z+1}$, $\Omega _{Y+1, X+1, Z}$, $\Omega _{Y, X+1, Z+1}$ and $\Omega _{Y, X+1, Z}$, the principle is also same as $\Omega _{Y, X, Z}$. The complexity of oracle operator $\Omega _{Y, X, Z}$ is $O((p+q)(h+w+l))$. The scaling-up circuit of 3-D floating-point data includes eight oracle operators of $\Omega _{Y+1, X, Z+1}$, $\Omega _{Y+1, X, Z}$, $\Omega _{Y, X, Z+1}$, $\Omega _{Y, X, Z}$, $\Omega _{Y+1, X+1, Z+1}$, $\Omega _{Y+1, X+1, Z}$, $\Omega _{Y, X+1, Z+1}$ and $\Omega _{Y, X+1, Z}$. Here, the total quantum cost in eight oracle operators is $O(8(p+q)(h+w+l))$. The network complexities of *Q*-*Adder* and *Q*-*Multiplier* modules are $O(p^2+q^2)$ and $O(p^3+q^2)$ based on^31,33,34^, respectively. According to Fig. 14, the *Converter* module can be decomposed into $(2^{p-1}-1)$ *adding* *one*, $(n-k)$ *k*-Control-*adding*-*one*, $k=1,2, \ldots , n-1$, $(n-1)$ Toffoli gates, $(n-m+1)$ *m*-controlled NOT gates, $m=3,4, \ldots , n$. The quantum cost of $(n-m+1)$ *m*-controlled NOT gates $(m=3,4, \ldots , n)$ is

$$\begin{aligned}&(n-3+1)(12\times 3-11)+(n-4+1)(12\times 4-11)+\ldots +(n-n+1)(12\times n-11)\\&\quad =2n^3+0.5n^2-4.5n-9. \end{aligned}$$

Only $(4k-8)$ 2-Control-Unitary gates were needed to construct 1 *k*-Control-Unitary gate, as well as some assistant qubits^34^. Therefore, 1 *k*-Control-*adding*-*one* can be constructed by $(4k-8)$ 2-Control-*adding*-*one*. The quantum cost of $(n-k)$ *k*-Control-*adding*-*one* $(k=1,2, \ldots , n-1)$ is

$$\begin{aligned}&(n-1)(4\times 1-8)p^2+ (n-2)(4\times 2-8)p^2+\ldots +[n-(n-1)][4\times (n-1)-8]p^2\\&\quad =p^2\left( \frac{2}{3}n^3-4n^2+\frac{10}{3}n\right) . \end{aligned}$$

To sum up, the complexity of the *Converter* module is $O((2^{p-1}-1)p^2+2n^3+0.5n^2-4.5n-9+6(n-1)+p^2(\frac{2}{3}n^3-4n^2+\frac{10}{3}n))\approx O(2^{p-1}p^2+n^3p^2)$.

*Case 1: The complexity of the scaling-up circuit.*

We analyze the complexity of the scaling-up circuit of 3-D floating-point data. The quantum circuit consists of 12 *Special* *Subtractor*, 8 *adding* *one*, 16 *Multiplier*, 8 *Q*-*Multiplier*, 7 *Q*-*Adder*, $8(h+w+l+h_1+w_1+l_1)$ CNOT gates, 8 oracle operators, 8 *Converter*, and $(h_1+w_1+l_1)$ *Divided* *by* 2.

Therefore, the complexity of the scaling-up circuit of 3-D floating-point data can be calculated as follows:

$$\begin{aligned} O\left( \begin{matrix} 4(h_1^2+w_1^2+l_1^2)\\ 4h^2+w^2+3l^2\\ 2(n_1^3+n_2^3+n_3^3+n_4^3+n_5^3+n_6^3+n_7^3+n_8^3)\\ 8(p^3+q^2)\\ 7(p^2+q^2)\\ 8(h+w+l+h_1+w_1+l_1)\\ 8(p+q)(h+w+l)\\ 8 p^2 2^{p-1}+(n_1^3+n_2^3+n_3^3+n_4^3+n_5^3+n_6^3+n_7^3+n_8^3)p^2\\ p^2(h_1+w_1+l_1) \end{matrix} \right) \approx O(p^2\alpha ^3+p^3+\beta ^2+q^2), \end{aligned}$$

where $$\alpha =\max (n_1,n_2,n_3,n_4,n_5,n_6,n_7,n_8)$$, $$\beta =\max (h, w, l)$$.

*Case 2: The complexity of the scaling-down circuit.*

Similar to the complexity of the scaling-up circuit, we analyze the complexity of the scaling-down circuit of 3-D floating-point data. The quantum circuit consists of 12 *Special* *Subtractor*, 12 *adding* *one*, 16 *Multiplier*, 8 *Q*-*Multiplier*, 7 *Q*-*Adder*, $8(h+w+l+h_1+w_1+l_1)$ CNOT gates, 8 oracle operators, 8 *Converter*, and $(h_1+w_1+l_1)$ *Divided* *by* 2.

Therefore, the complexity of the scaling-down circuit of 3-D floating-point data can be calculated as follows:

$$\begin{aligned} O\left( \begin{matrix} 4(h_1^2+w_1^2+l_1^2)\\ 4(h^2+w^2+l^2)\\ 2(n_1^3+n_2^3+n_3^3+n_4^3+n_5^3+n_6^3+n_7^3+n_8^3)\\ 8(p^3+q^2)\\ 7(p^2+q^2)\\ 8(h+w+l+h_1+w_1+l_1)\\ 8(p+q)(h+w+l)\\ 8 p^2 2^{p-1}+(n_1^3+n_2^3+n_3^3+n_4^3+n_5^3+n_6^3+n_7^3+n_8^3)p^2\\ p^2(h_1+w_1+l_1) \end{matrix} \right) \approx O(p^2\alpha ^3+p^3+\beta ^2+q^2), \end{aligned}$$

where $$\alpha =\max (n_1,n_2,n_3,n_4,n_5,n_6,n_7,n_8)$$, $$\beta =\max (h, w, l)$$.

**Figure 17:**
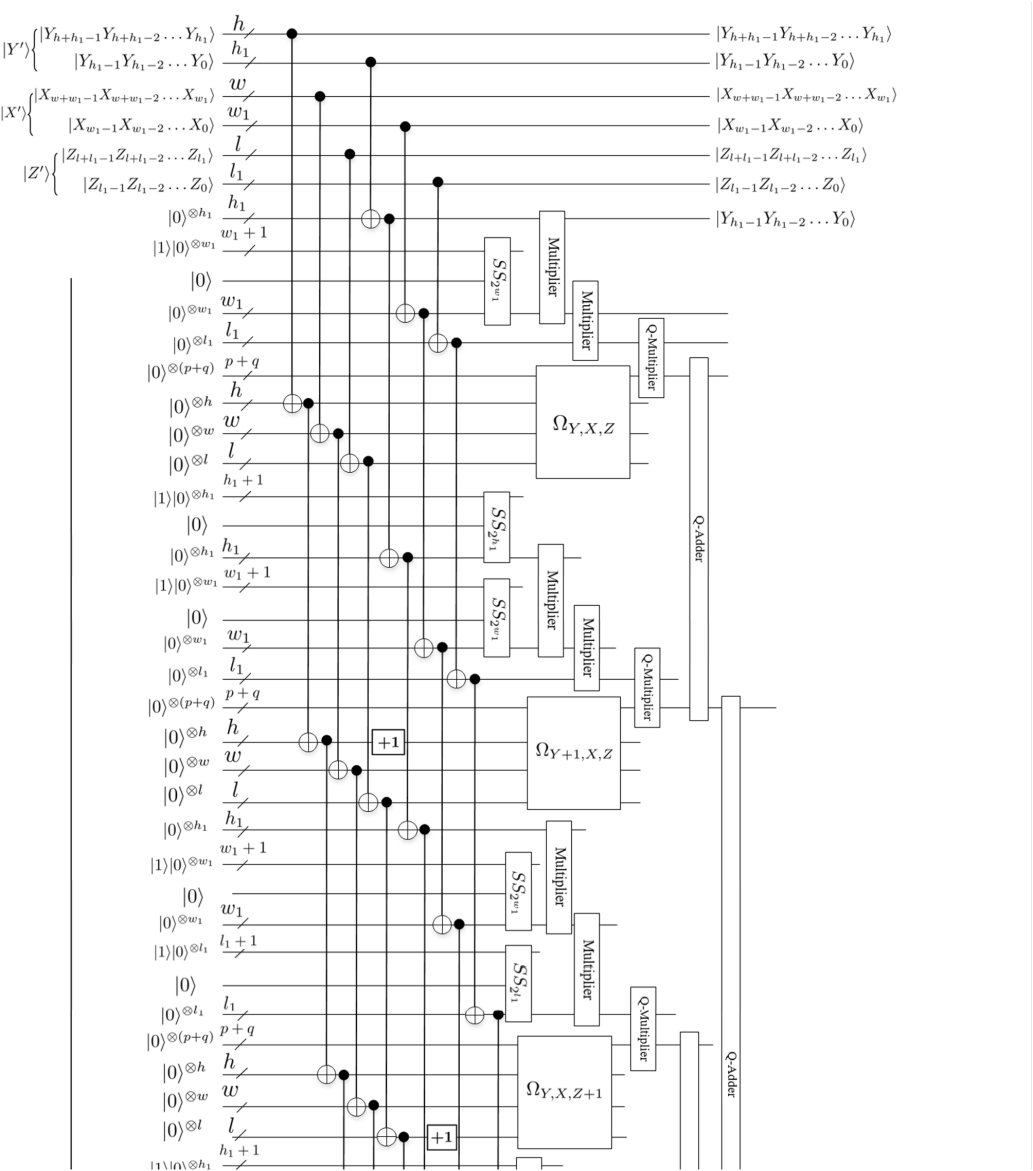
Scaling-up circuit(1).

**Figure 18:**
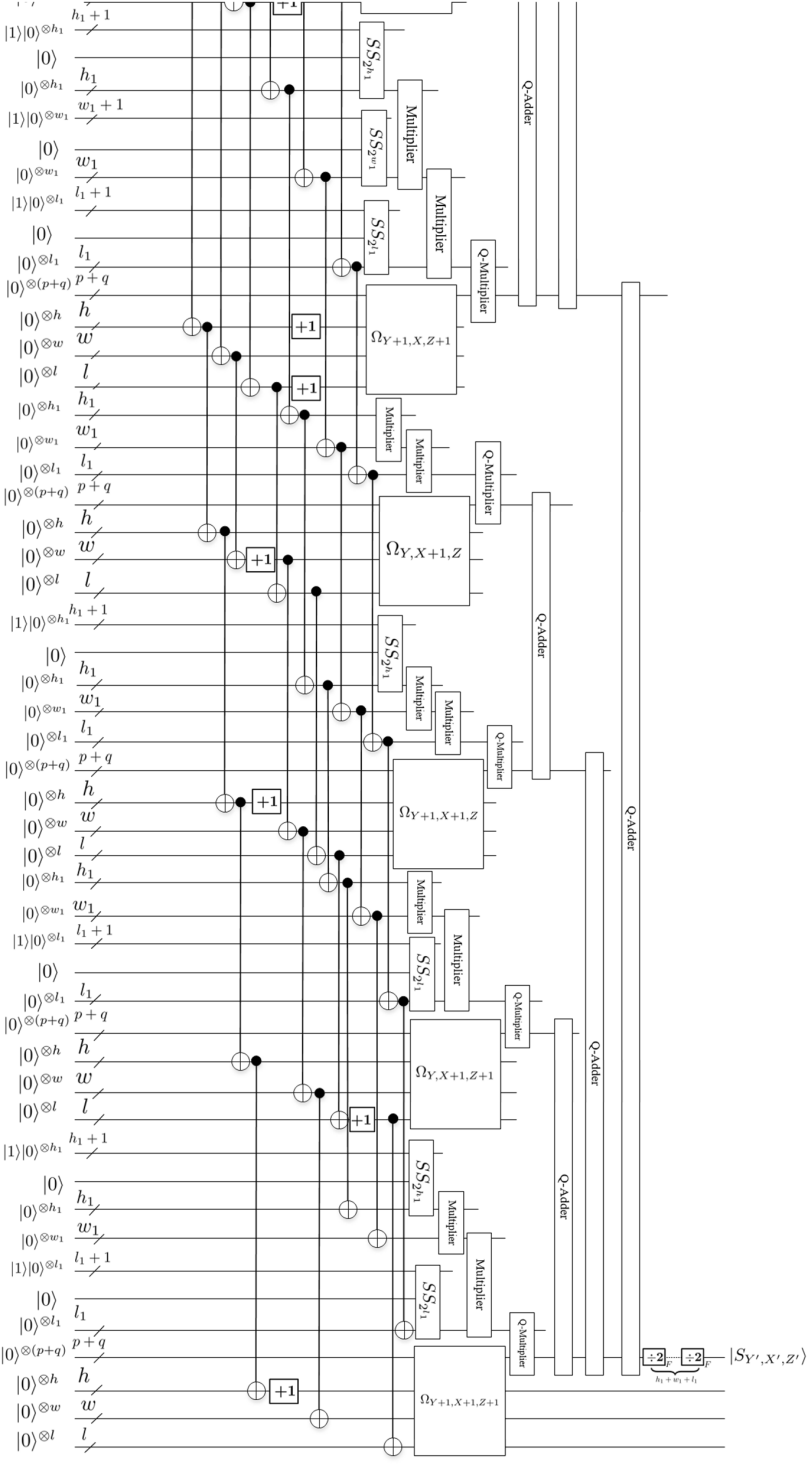
Scaling-up circuit(2).

**Figure 19:**
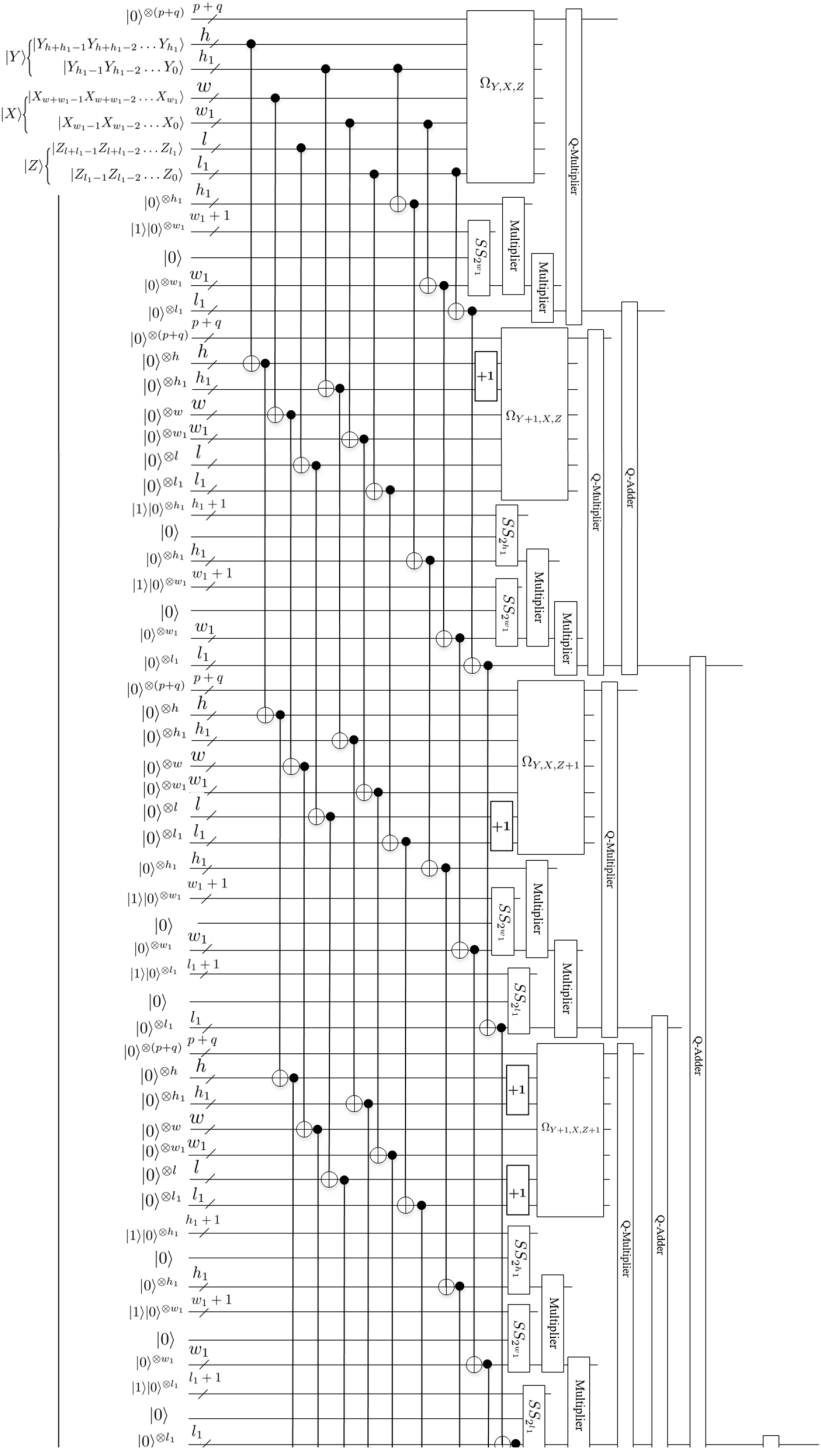
Scaling-down circuit(1).

**Figure 20:**
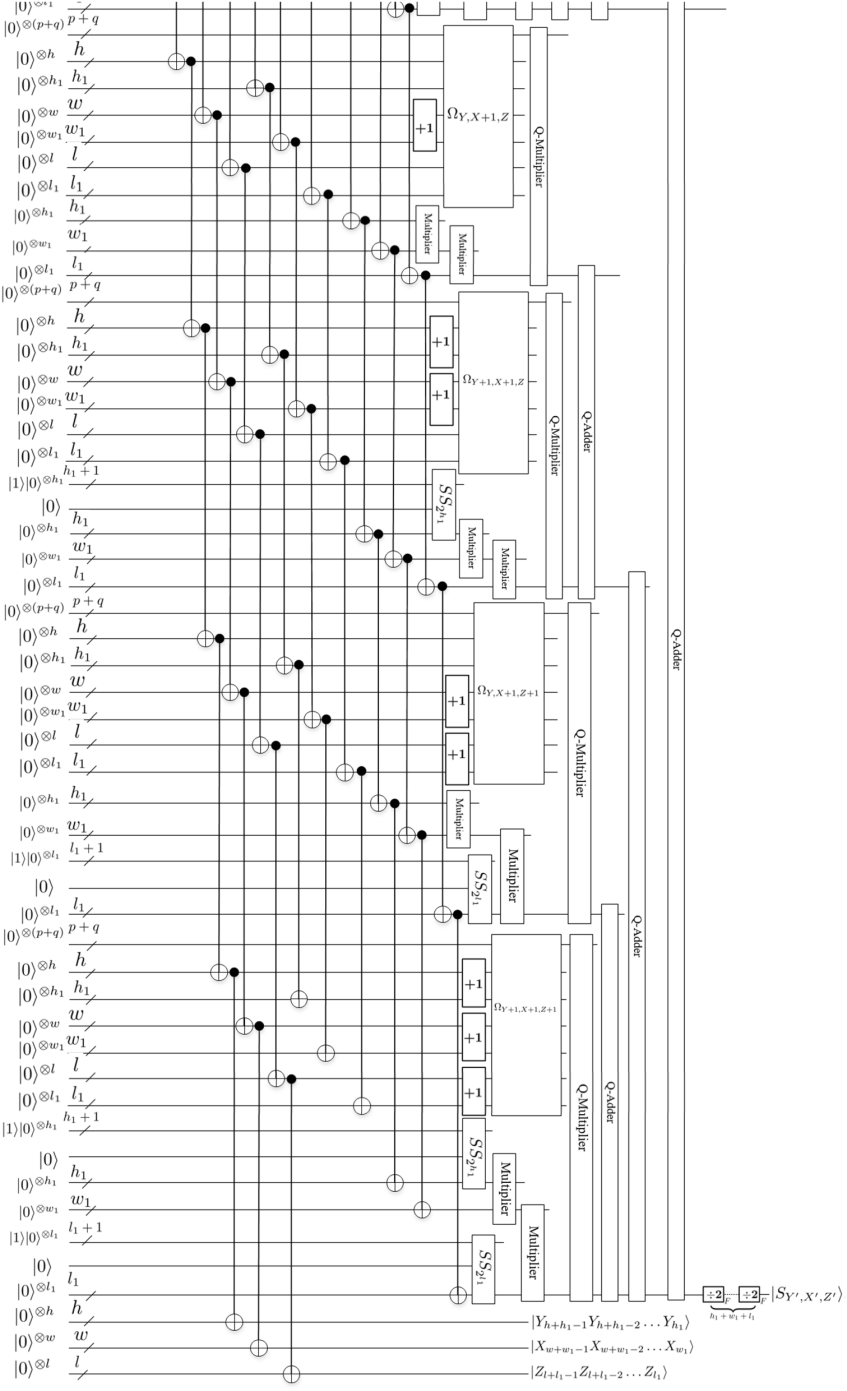
Scaling-down circuit(2).

## Conclusions

Quantum computation has become a novel and important tool in the field of image/data processing. In this paper, the trilinear interpolation method for quantum scaling up and down of 3-D floating-point data is proposed for the first time. 3-D data have a wide range of applications in many diverse fields such as artificial intelligence, aeronautics, architecture, biological science, medicine, etc. Floating-point numbers offer great savings in the number of qubits when the required range of values and/or relative precision is large. Therefore, based on QFT, we have designed the addition and multiplication (Q-Adder and Q-Multiplier modules) of 3-D floating-point data. And then, we have proposed a Converter module for converting fixed-point numbers to floating-point numbers. Combining some basic modules in this paper, we can achieve the quantum scaling up and down for 3-D floating-point data. Finally, we have proposed the design scheme of quantum scaling up and down for 3-D floating-point data using trilinear interpolation method based on QFT.

In future work, the design scheme of quantum scaling up and down is extremely helpful for quantum reconstruction theory, such as the reconstruction technology of 2-D to 3-D data. And we can use the reconstruction technology to perform quantum data processing such as medical data reconstruction in future research.

## References

[1] Feynman RP (1982). Simulating physics with computers. Int. J. Theor. Phys..

[2] Shor, P. W. Algorithms for quantum computation: Discrete logarithms and factoring. In *Proceedings 35th Annual Symposium on Foundations of Computer Science* 124–134 (1994).

[3] Grover, L. K. A fast quantum mechanical algorithm for database search. In *Proceedings of the 28th Annual ACM Symposium on the Theory of Computing* 212–219 (1996).

[4] Venegas-Andraca, S. E. & Bose, S. Storing, processing and retrieving an image using quantum mechanics. In *Proceeding of SPIE Conference of Quantum Information and Computation 5105* 137–147 (2003).

[5] Latorre, J. I. Image compression and entanglement. *Computer. Science* 1–4 (2005).

[6] Venegas-Andraca SE, Ball JL (2010). Processing images in entangled quantum systems. Quantum Inf. Process..

[7] Le PQ, Dong F, Hirota K (2011). A flexible representation of quantum images for polynomial preparation, image compression, and processing operations. Quantum Inf. Process..

[8] Zhang Y, Lu K, Gao Y, Wang M (2013). NEQR: a novel enhanced quantum representation of digital images. Quantum Inf. Process..

[9] Jiang N, Wang L (2015). Quantum image scaling using nearest neighbor interpolation. Quantum Inf. Process..

[10] Jiang N, Wang J, Mu Y (2015). Quantum image scaling up based on nearest-neighbor interpolation with integer scaling ratio. Quantum Inf. Process..

[11] Li H, Fan P, Xia H, Peng H, Song S (2019). Quantum implementation circuits of quantum signal representation and type conversion. IEEE Trans. Circuits Syst. I Regul. Pap..

[12] Zhang R, Lu D, Yin H (2020). A generalized floating-point representation and manipulation of quantum signals based on IEEE-754. Int. J. Theor. Phys..

[13] Zhang R, Xu M, Lu D (2020). A generalized floating-point quantum representation of 2-D data and their applications. Quantum Inf. Process..

[14] Chetia R, Boruah SMB, Sahu PP (2021). Quantum image edge detection using improved Sobel mask based on NEQR. Quantum Inf. Process..

[15] Chakraborty, S., Mandal, S. B. & Shaikh, S. H. Quantum image processing: challenges and future research issues. *Int. J. Inf. Technol.* 1–15 (2018).

[16] S. Chakraborty, S. B. Mandal, S. H. Shaikh, and L. Dey. Ternary quantum circuit for color image representation. In *Advanced Computing and Systems for Security* 95–108 (2017).

[17] Chakraborty S, Mandal SB, Shaikh SH (2018). Design and implementation of a multivalued quantum circuit for threshold based color image segmentation. Intell. Decis. Technol..

[18] Fijany, A. & Williams, C. P. Quantum wavelet transforms: fast algorithms and complete circuits. In *NASA international conference on quantum computing and quantum communications* 10–33 (1998).

[19] Caraiman S, Manta V (2013). Quantum image filtering in the frequency domain. Adv. Electr. Comput. Eng..

[20] Ruiz-Perez L, Garcia-Escartin JC (2017). Quantum arithmetic with the quantum Fourier transform. Quantum Inf. Process..

[21] Li P, Sun H (2018). Quantum color image filtering in the frequency domain. J. Electron. Inf. Technol..

[22] Asaka R, Sakai K, Yahagi R (2020). Quantum circuit for the fast Fourier transform. Quantum Inf. Process..

[23] Chakraborty S, Shaikh SH, Chakrabarti A, Ghosh R (2020). An image denoising technique using quantum wavelet transform. Int. J. Theor. Phys..

[24] Chakraborty, S., Shaikh, S. H., Chakrabarti, A. & Ghosh, R. A study of scrambled noisy quantum image formation with geometric transformation and its denoising using QWT. In *High Performance Vision Intelligence: Recent Advances* 137–150 (2020).

[25] Chang WL, Vasilakos AV (2021). Fundamentals of Quantum Programming in IBM’s Quantum Computers.

[26] Grigoryan AM, Agaian SS (2020). New look on quantum representation of images: Fourier transform representation. Quantum Inf. Process..

[27] Sang J, Wang S, Niu X (2016). Quantum realization of the nearest-neighbor interpolation method for FRQI and NEQR. Quantum Inf. Process..

[28] Zhou R, Hu W, Fan P, Ian H (2017). Quantum realization of the bilinear interpolation method for NEQR. Sci. Rep..

[29] Li P, Liu X (2018). Bilinear interpolation method for quantum images based on quantum Fourier transform. Int. J. Quantum Inf..

[30] Zhou R, Cheng Y, Liu D (2019). Quantum image scaling based on bilinear interpolation with arbitrary scaling ratio. Quantum Inf. Process..

[31] Nielsen MA, Chuang IL (2000). Quantum Computation and Quantum Information.

[32] Zhang Y, Lu K, Xu K, Gao Y, Wilson R (2015). Local feature point extraction for quantum images. Quantum Inf. Process..

[33] Haener, T., Soeken, M., Roetteler, M. & Svore, K. M. Quantum circuits for floating-point arithmetic. *Lect. Notes Comput. Sci.* 162–174 (2018).

[34] Barenco A, Bennett CH, Cleve R, Divincenzo DP, Margolus N, Shor P, Sleator T, Smolin J, Weinfurter H (1995). Elementary gates for quantum computation. Phys. Rev. A.

